# *Mycobacterium abscessus* pathogenesis identified by phenogenomic analyses

**DOI:** 10.1038/s41564-022-01204-x

**Published:** 2022-08-25

**Authors:** Lucas Boeck, Sophie Burbaud, Marcin Skwark, Will H. Pearson, Jasper Sangen, Andreas W. Wuest, Eleanor K. P. Marshall, Aaron Weimann, Isobel Everall, Josephine M. Bryant, Sony Malhotra, Bridget P. Bannerman, Katrin Kierdorf, Tom L. Blundell, Marc S. Dionne, Julian Parkhill, R. Andres Floto

**Affiliations:** 1 Molecular Immunity Unit, University of Cambridge Department of Medicine, MRC Laboratory of Molecular Biology, Cambridge, UK; 2 Cambridge Centre for AI in Medicine, Cambridge, UK; 3 Wellcome Sanger Institute, Hinxton, UK; 4 Department of Biomedicine, University of Basel, Basel, Switzerland; 5 Department of Biochemistry, University of Cambridge, Cambridge, UK; 6 MRC Centre for Molecular Bacteriology and Infection, Imperial College London, London, UK; 7 Department of Life Sciences, Imperial College London, London, UK; 8 Scientific Computing Department, Science and Technology Facilities Council, Harwell, UK; 9 Institute of Neuropathology, Faculty of Medicine, University of Freiburg, Freiburg, Germany; 10 Department of Veterinary Medicine, University of Cambridge, Cambridge, UK; 11 Cambridge Centre for Lung Infection, Royal Papworth Hospital, Cambridge, UK

**Keywords:** Bacterial genetics, Genetic linkage study

## Abstract

The medical and scientific response to emerging and established pathogens is often severely hampered by ignorance of the genetic determinants of virulence, drug resistance and clinical outcomes that could be used to identify therapeutic drug targets and forecast patient trajectories. Taking the newly emergent multidrug-resistant bacteria *Mycobacterium abscessus* as an example, we show that combining high-dimensional phenotyping with whole-genome sequencing in a phenogenomic analysis can rapidly reveal actionable systems-level insights into bacterial pathobiology. Through phenotyping of 331 clinical isolates, we discovered three distinct clusters of isolates, each with different virulence traits and associated with a different clinical outcome. We combined genome-wide association studies with proteome-wide computational structural modelling to define likely causal variants, and employed direct coupling analysis to identify co-evolving, and therefore potentially epistatic, gene networks. We then used in vivo CRISPR-based silencing to validate our findings and discover clinically relevant *M. abscessus* virulence factors including a secretion system, thus illustrating how phenogenomics can reveal critical pathways within emerging pathogenic bacteria.

## Abstract (web summary)

Antibiotic resistance and virulence factors are identified in *Mycobacterium abscessus* by integrating proteome-wide structural modelling, GWAS analyses and mapping gene interaction networks for 331 clinical isolates.

## Main

Over the past two decades, *Mycobacterium abscessus*, a rapidly growing species of non-tuberculous mycobacteria, has emerged as a major threat to individuals with cystic fibrosis (CF) and other chronic lung disease^1^. Rates of infection of CF patients have increased around the world^1,2^, due to unknown factors, potentially including hospital-based person-to-person transmission^3,4^ and the emergence of globally spread dominant circulating clones that are associated with increased virulence and worse clinical outcomes^5^. Infections with *M. abscessus* are challenging and sometimes impossible to treat^1,6,7^, lead to accelerated inflammatory lung damage^8,9^ and may prevent safe transplantation^10^. To date, very little is known about how *M. abscessus* infects humans, how it causes inflammatory lung damage and how it resists antibiotics^11^. There is thus an urgent need to better understand the pathophysiology of *M. abscessus*, define optimal drug targets and predict the virulence and antibiotic susceptibility of clinical isolates.

Historically, systems-level approaches to understanding the genetic determinants of bacterial behaviour have been limited to evaluating the phenotypes of experimentally created mutant libraries^12^. However, advances in whole-genome sequencing now allow large-scale capture of the genetic and phenotypic diversity of clinical isolates and, consequently, the use of genome-wide association studies (GWAS) to define potentially causal variants.

Bacterial GWAS analyses have been successfully deployed to identify genetic determinants of antibiotic resistance^13^ and virulence^14^, but could potentially be used for any heritable bacterial trait. There are, however, several factors that limit the application of GWAS approaches to bacteria including: the complex correlations and interdependencies of phenotypes, obscuring causality; the presence of genome-wide linkage disequilibrium leading to ambiguity over which variant is causal, necessitating accurate modelling of the functional impacts of mutations; and the fact that most bacterial phenotypes are complex traits, not explained by monogenetic features, but rather functional interactions of larger groups of proteins. To advance our pathophysiological understanding of bacteria, we therefore need to discover both comprehensive sets of causal genetic variants and complex gene–gene (or ‘epistatic’) interactions.

We sought to combine detailed in vitro and in vivo phenotyping, whole-genome sequencing, computational structural modelling and epistatic analysis to provide a phenogenomic map of *M. abscessus* that might define critical pathways involved in virulence and drug resistance.

## Results

### Multidimensional phenotyping in *M. abscessus*

**Fig. 1:**
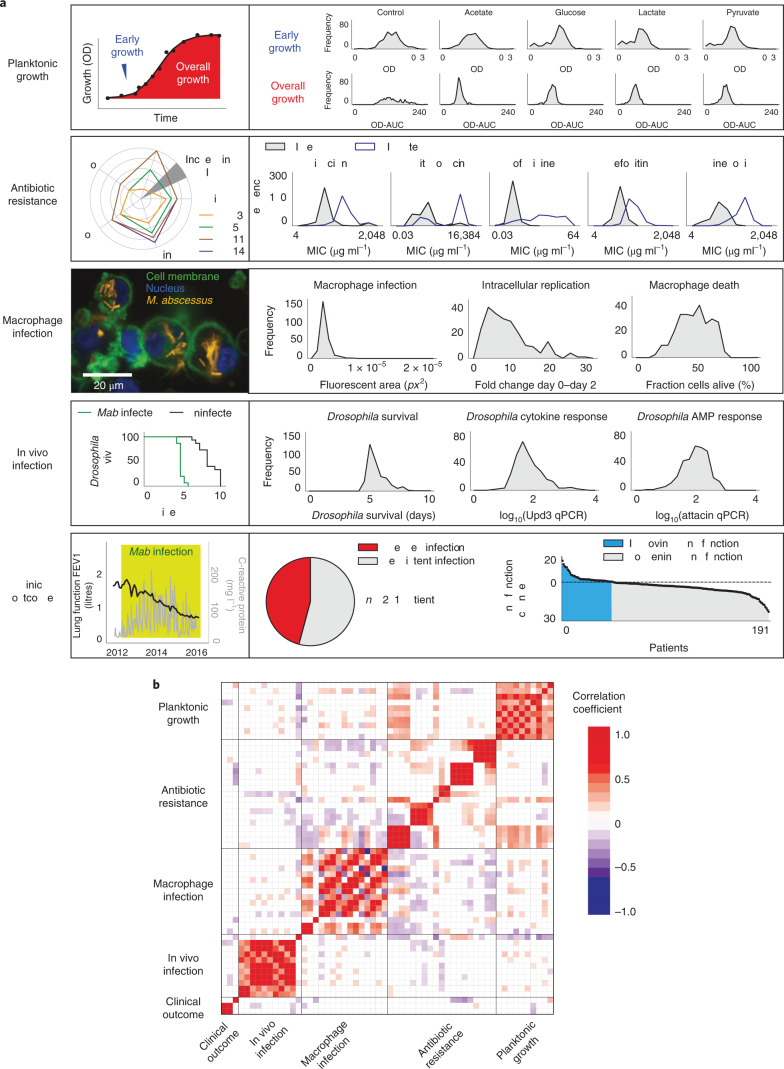
Multidimensional phenotyping of *M. abscessus*. **a**, Phenotypic variability of clinical *M. abscessus* isolates was assessed across multiple dimensions (described in Methods) including: planktonic growth (assessed by serial OD measurement) in a range of different carbon sources; MIC of a range of clinically relevant antibiotics assessed on day 3 (MIC early) and day 11 (MIC late) to quantify intrinsic and inducible drug resistance; macrophage infection (4 h post infection), intracellular replication (2 days post infection) and death (2 days post infection) quantified using high-content imaging of differentiated THP-1 cells incubated with tdTomato-expressing clinical isolates; survival and immune response of *Drosophila melanogaster* infected with clinical isolates; and clinical outcomes (lung function decline and clearance of *M. abscessus* from sputum samples) of infected patients. Ami, amikacin; Cla, clarithromycin; Clo, clofazimine; FEV1, forced expiratory volume; Fox, cefoxitin; Lin, linezolid; OD-AUC, area under the OD curve; qPCR, quantitative polymerase chain reaction. **b**, Pearson correlation coefficients within and across phenotypic groups shown as a matrix, with two-sided non-significant (unadjusted *P* > 0.05) associations shown in white. Source data

We first characterized 331 clinical *M. abscessus* isolates across 58 phenotypic dimensions exploring five key pathogenic traits: planktonic growth in different carbon sources; antibiotic resistance (at early and late time points) against a selection of drugs recommended by clinical treatment guidelines^1^; in vitro infection of a human macrophage cell line model (differentiated THP-1 cells), monitored using high-content confocal microscopy; in vivo infection of *Drosophila melanogaster*, measuring host survival and inflammatory responses; and clinical outcomes following infection, available through previously collected metadata^5^ (Fig. 1a and Supplementary Figs. 1 and 2).

**Fig. 2:**
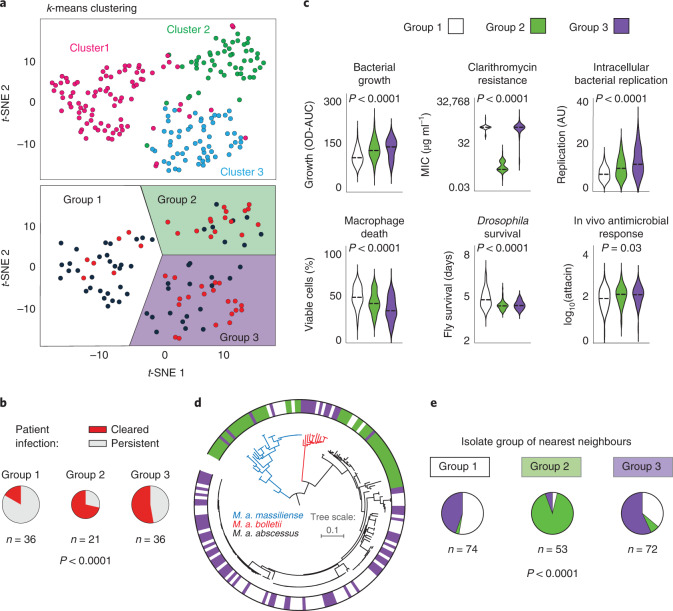
Phenotypic groups. **a**,**b**, Clustering of clinical isolates, using *k*-means (pink, blue, green) or *t*-SNE, based on experimentally observed phenotypes only, reveals similar groups that differ in their clinical outcomes. **c**, Distribution of specific phenotypes across the three phenotypic groups (bacterial growth: *P* = 3.6 × 10^−7^; clarithromycin resistance: *P* = 2.5 × 10^−115^; intracellular bacterial replication *P* = 1.8 × 10^−10^; macrophage death: *P* = 5.1 × 10^−10^; *Drosophila* survival: *P* = 8.5 × 10^−11^). AU, arbitrary units. **d**, Maximum likelihood phylogenetic tree of *M. abscessus* subspecies and corresponding phenotypic groups. **e**, Nearest phylogenetic neighbours most commonly belong to the same phenotypic group. *P* values were calculated using a two-tailed chi-squared test or one-way analysis of variance, as appropriate. Source data

We examined the relationship between phenotypes, finding correlations within, and sometimes between, pathogenic traits (Fig. 1b and Supplementary Fig. 3). To explore whether there were distinct patterns of bacterial behaviours, we used experimentally derived data to plot individual isolates in phenotypic space, identifying three discrete groups, each associated with different clinical outcomes (Fig. 2a–c and Supplementary Fig. 3). Specific phenotypic groups were overrepresented in particular clades and among phylogenetic nearest neighbours, indicating that these phenotypic groups represent distinct heritable traits (Fig. 2d,e).

Isolates from Group 3 demonstrated the fastest growth in liquid culture and quickest replication within macrophages, caused higher mortality in infected macrophages and *Drosophila*, and the greatest antimicrobial and inflammatory responses in flies, whereas Group 1 isolates had the opposite characteristics. Group 2 isolates had phenotypic behaviours that were intermediate compared with the other two groups and were associated with the most favourable clinical outcome, potentially related to their macrolide susceptibility (a key determinant of treatment response^15,16^) explained by known *erm41* and 23S ribosomal RNA genotypes (Supplementary Fig. 3). By contrast, we found that, despite having similar levels of macrolide resistance, Group 1 and Group 3 isolates were associated with very different clinical outcomes in infected patients, highlighting the importance of phenotypic characteristics other than antimicrobial susceptibility in determining prognosis, and suggesting that immunogenic isolates might be cleared more easily by patients (as reported previously for other pathogenic bacteria^17–20^).

We next examined the contribution of different colony morphotypes and *M. abscessus* subspecies to the phenotypic analysis. Although morphotype transition from smooth to rough, caused by disrupted glycopeptidolipid production, has previously been linked to increased in vitro and in vivo virulence^11,21^, the 18% of our isolates that were of the rough morphotype were not associated with worse patient outcomes, or changes in outcome during macrophage or *Drosophila* infection (Supplementary Fig. 4). Similarly, stratifying by *M. abscessus* subspecies revealed no differences in clinical outcome and only limited differences in phenotypic behaviour (apart from the expected difference in clarithromycin resistance due to recognized *erm41* truncation in *M. abscessus* subspecies *massiliense;* Supplementary Fig. 4). Phenotypic clustering and resultant group composition were not affected by considering only isolates with a smooth morphotype or from the *M. a. abscessus* subspecies, indicating that our analysis has uncovered unexpected phenotypic relationships.

### Structure-guided GWAS

**Fig. 3:**
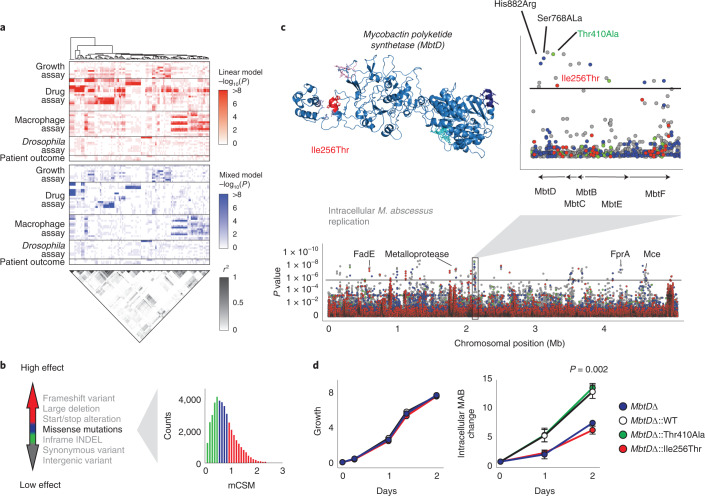
Integrating computational structural modelling into GWAS. **a**, Genome-wide associations were performed for all phenotypes with the top variants extracted (up to five per association) and ordered using hierarchical clustering (red, linear model; blue, mixed model; *P* values were calculated using a two-sided Wald test). Pairwise *R*^2^ measurements of the identified genetic variants (grey scale) show extensive genome-wide linkage (LD). **b**, To identify causal variants and overcome genome-wide linkage, the functional impacts of genetic variants were classified as having high effects (large deletions, frameshifts, start/stop alterations; red), moderate effects (inframe insertions/deletions; blue and green) and low effects (synonymous and intergenic variants; grey). The impacts of missense mutations were estimated using proteome-wide computational structural modelling with variants considered as having high (red), moderate (blue) or low (green) functional effects based on terciles of the change in protein stability, estimated using mCSM. **c**, Manhattan plot of the mixed model GWAS analysis of 264,122 genetic variants for intracellular *M. abscessus* replication (two-sided Wald test) with the threshold for multiple hypothesis testing (black line). Several loci in *mbtD*, including four missense mutations, were identified as potential mechanisms relevant for intracellular *M. abscessus* survival. (Inset) Three-dimensional structural model of MbtD with the high-effect missense mutation Ile256Thr shown in red. **d**, *MbtD* knockout mutants complemented with wild-type or identified *MbtD* variants had similar growth rates in broth culture but replicated differently within THP-1 cells. Experiments were performed in triplicate on at least three separate occasions and are presented as mean ± s.e.m. Conditions were compared with a two-sided unpaired *t-*test. Source data

To understand the genetic basis for these important variations in *M. abscessus* behaviour, we used whole-genome sequence data to perform a GWAS for each phenotype (Fig. 3a), evaluating approximately 270,000 genetic variants comprising single nucleotide polymorphisms (SNPs), insertions and deletions (INDELs). We used mixed models corrected for population structure^22^ to identify locus effects, as well as uncorrected linear models to ensure we captured lineage effects^23^. In total, we identified 1,926 hits (involving 1,000 genes) across 46 phenotypes (Supplementary Data). These included previously known genetic determinants, such as the 16S and 23S rRNA mutations associated with constitutive aminoglycoside and macrolide resistance (*P* = 1.3 × 10^−75^ and *P* = 1.5 × 10^−54^ respectively; Supplementary Fig. 5), thereby confirming the effectiveness of our approach.

Current GWAS approaches are limited in their ability to accurately identify causal variants by both the presence of linkage disequilibrium, which in the case of *M. abscessus* (as with other bacteria^24,25^) is extensive and genome-wide (Fig. 3a and Supplementary Fig. 6), and by a failure to consider the impact of mutations on protein function^26,27^.

We therefore applied proteome-wide computational structural modelling to evaluate the probable functional impact of all non-synonymous SNPs across the genome, by applying our graph-based machine learning method mutation cut-off scanning matrix (mCSM)^28^ to our comprehensive *M. abscessus* structural database *Mabellini*^29^ (Fig. 3b) to identify probably causal mutations.

As an example, the GWAS for intracellular replication of *M. abscessus* within macrophages identified a number of hits at genome-wide significance including a cluster of variants within mycobactin synthesis genes (Fig. 3c). Mycobactins are mycobacterially produced iron chelators that efficiently scavenge iron during intracellular growth within macrophages, providing the iron essential for mycobacterial protein synthesis and other critical cell processes^30,31^. Structural modelling predicted that one variant, a missense mutation (Ile256Thr) in the mycobactin polyketide synthetase (*mbtD*) gene, was most likely to result in loss of protein function and therefore be causally related to the phenotypic change, probably through altering the ability of intracellular *M. abscessus* to access iron. To experimentally validate this structural modelling, we created an *MbtD* knockout mutant that demonstrated impaired intracellular growth in macrophages, and was able to be complemented by episomal expression of MbtD with the Thr410Ala mutation (predicted by mCSM to be tolerated), but not by the Ile256Thr mutation (predicted to be deleterious; Fig. 3d).

### Analysis of genome-wide epistasis through mutational co-evolution

**Fig. 4:**
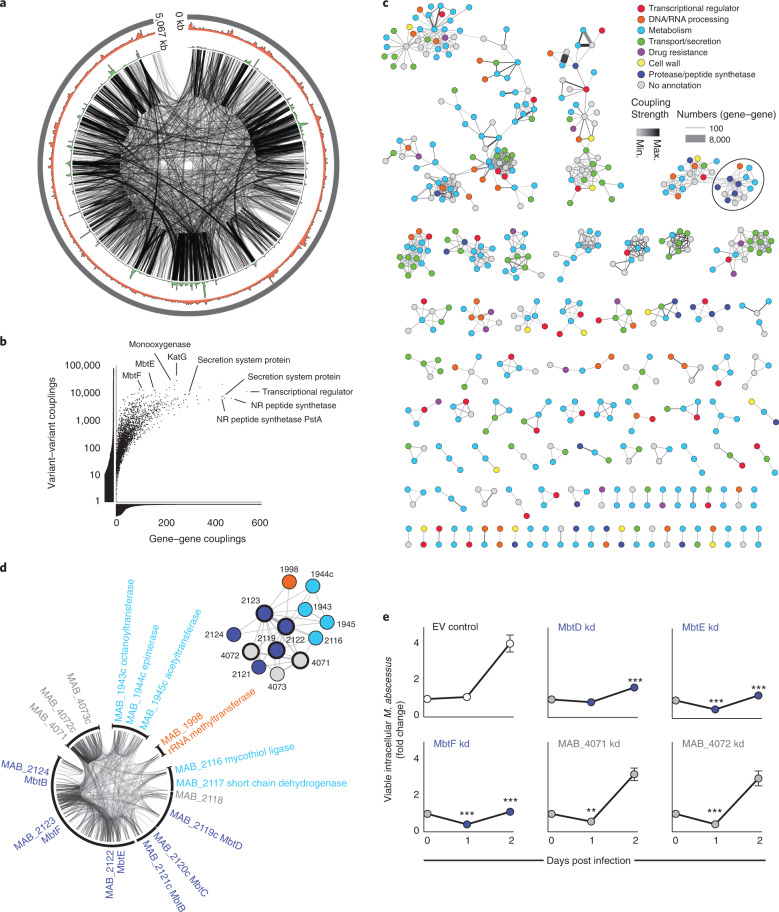
CC-DCA for assessing genome-wide epistasis. CC-DCA was used to identify co-evolving variants among ~10^12^ potential variant combinations of 2,366 clinical *M. abscessus* isolates. **a**, Circos plot of the *M. abscessus* chromosome showing the 100,000 top variant-to-variant couplings with a distance of >100 bp (black lines), coupling density (green; range 0–56,307 couplings per 5 kb) and SNP density (red; range 14–1,961 SNPs per 5 kb). **b**, Significant variant–variant couplings identified through DCA were pooled to gene–gene couplings. Whereas variant–variant couplings indicate the total number of co-evolutionary signals within a single gene, gene–gene couplings reflect the number of putative gene interactions. NR, non-ribosomal. **c**, Networks of co-evolving (and therefore probably epistatic) genes based on the 1,000 strongest DCA-derived gene–gene couplings ranked by coupling number, colour coded by functional class. The strength and number of couplings are shown by edge colour and thickness respectively. **d**, Example of a highly coupled gene network (highlighted by a circle in **c**) involving components of the mycobactin biosynthesis pathway **e**, CRISPR-induced transcriptional repression of several genes within this cluster demonstrates impaired mycobacterial survival within macrophages. Experiments were done in triplicate (with three guides per gene) on at least three separate occasions, are presented as mean ± s.e.m., and compared with the empty vector control using a two-sided unpaired *t*-test, **P < 0.001, ***P < 0.0001 (MbtD knockdown d2: *P* = 0.0002; MbtE kd d1: *P* = 0.0006, d2: *P* = 4.3 × 10^−5^; MbtF kd d1: *P* = 0.0001, d2: *P* = 1.3 × 10^−5^; MAB_4071 kd d1: *P* = 0.008; MAB_4072 kd d1: *P* = 0.0002). Source data

To understand whether mutations across the genome might have co-evolved, indicating potential epistatic interactions between genes, we deployed correlation-compressed direct coupling analysis (CC-DCA^32^) on whole-genome sequences from 2,366 clinical isolates of *M. abscessus* to identify whether variant co-occurrence deviated from the expected frequencies based on linkage disequilibrium^33,34^, and thus indicates evolutionary co-selection. We evaluated 10^12^ potential couplings (resulting from approximately 10^6^ genetic variants) and identified 1,168,913 that were significantly enriched (accepting a false discovery rate (FDR) of 10^−6^; Fig. 4a and Supplementary Fig. 6). We found many enriched couplings between known or predicted virulence genes (Fig. 4b and Supplementary Data), indicating pathogenic evolution of *M. abscessus* (as identified previously^5,35^). We used the ranked outputs from the CC-DCA analysis to establish discrete networks of genes that have co-evolved, and thus probably interact functionally (Fig. 4c). Many of these putative interactions could be recapitulated using orthogonal information provided by the STRING database (Supplementary Fig. 7)^36^. As examples, we find highly connected clusters of mammalian cell entry genes, implicated in controlling adhesion, uptake and intracellular survival within macrophages^37,38^, and genes involved in bacterial secretion systems. In addition, we discovered a network of mycobactin synthesis genes (Fig. 4d), including some identified through our GWAS analysis (Fig. 3c,d) that, when silenced by CRISPR interference (CRISPRi) knockdown, led to similar impairment of intracellular bacterial growth (Fig. 4e), supporting a functional basis for these CC-DCA-derived gene networks.

### Defining genetic determinants of in vivo virulence in *M. abscessus*

Finally, we sought to integrate outputs from our detailed multidimensional phenotyping, structure-guided GWAS analysis and DCA-based epistatic mapping, to achieve a systems-level understanding of the genetic basis for important pathological processes in *M. abscessus*.

**Fig. 5:**
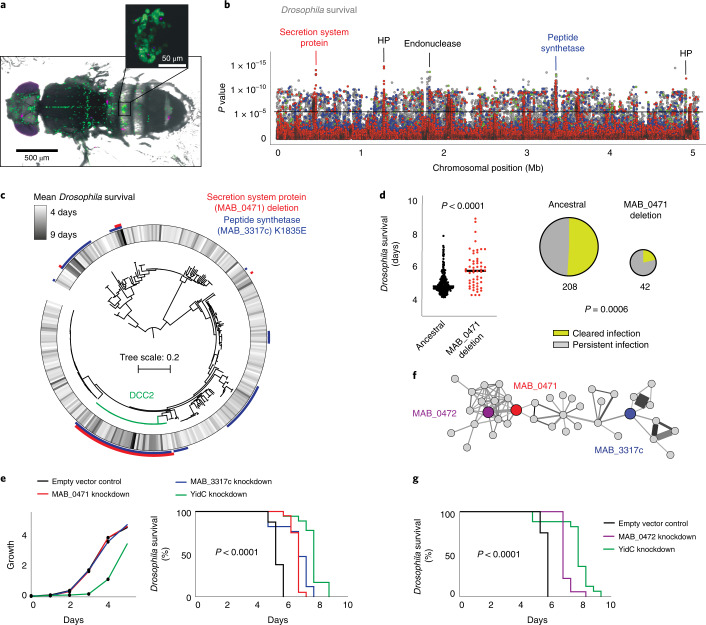
Integrating GWAS and DCA to reveal the genetic networks of in vivo virulence in *M. abscessus*. **a**, Representative image of *Drosophila melanogaster* infected with *M. abscessus* (magenta) resembles mycobacterial infection in other organisms (independently repeated over five times), with infection of phagocytes (green) and formation of granuloma-like structures (inset). **b**, Genome-wide association (using a linear model and applying Wald test statistics) reveals a putative secretion system protein and a peptide synthetase to be highly associated with *Drosophila* survival. The black horizontal line marks the multiple hypothesis testing threshold based on the number of independent variants. HP, hypothetical protein. **c**, Both variants align to clinical isolates with long survival, including a dominant circulating clone, within the subspecies *M. a. abscessus*. **d**, Deletion in *MAB_0471* was associated with persistent respiratory infection in CF patients (two-sided unpaired *t*-test). **e**, CRISPR–dCas9 knockdown of *MAB_0471* and *MAB_3317* (unlike the essential gene *yidC*) did not affect growth in liquid culture (left) but in vivo silencing did lead to prolonged survival of infected *Drosophila*, as shown by Kaplan–Meier survival analysis (log-rank test, *P* = 7.6 × 10^−17^) generated from data from at least 18 infected flies per bacterial strain. **f**, Epistatic gene network, derived from DCA outputs, revealed direct coupling of *MAB_0471* with other putative secretion system proteins including *MAB_0472* and a distant connection to the peptide synthetase *MAB_3317*. **g**, In vivo silencing of *MAB_0472* replicated virulence attenuation (log-rank test, *P* = 3.6 × 10^−12^). Source data

We focused on in vivo infection in *Drosophila*, a model that replicates some features of human mycobacterial infection (particularly innate and cell-autonomous immune responses) (Fig. 5a)^39–42^. Among the top hits from our structure-guided GWAS analysis (Fig. 5b and Supplementary Fig. 8) were a deletion in a component of a putative Type II secretion system (*MAB_0471*) and a deleterious mutation in a non-ribosomal peptide synthetase (*MAB_3317c*). Both variants had independently arisen as homoplastic mutations across the *M. abscessus* phylogenetic tree (Fig. 5c), including within the ancestor of one of the dominant circulating clones (DCC2) of *M. a. abscessus*, responsible for several transmission networks among CF patients^3,5^. We found that isolates with either of the two genetic variants were associated with prolonged survival of infected *Drosophila* and more persistent clinical infection of CF patients (Fig. 5d and Supplementary Fig. 8).

We sought to experimentally validate both these GWAS hits through CRISPRi-based transcriptional silencing as described previously^43^. Although we found no effect of gene silencing on growth in liquid media, silencing of either *MAB_0471* or *MAB_3317c* during in vivo infection significantly increased *Drosophila* survival (Fig. 5e and Supplementary Figs. 8 and 9), indicating that these genes regulate *M. abscessus* virulence.

Our DCA analysis revealed that both these GWAS hits were part of a discrete network of likely epistatic genes involved in bacterial secretion, cell wall biosynthesis, metabolism and transcriptional regulation (Fig. 5f and Supplementary Fig. 8). To experimentally test this predicted epistasis, we selected another gene from the same network (*MAB_0472*) and transcriptionally silenced it during in vivo infection. We found that *Drosophila* survival was also increased by its CRISPRi knockdown (Fig. 5g), suggesting that all three genes are functionally interacting.

## Discussion

We have shown that phenogenomic analysis can accurately identify critical gene networks responsible for virulence and other characteristics in poorly understood bacterial pathogens, such as *M. abscessus*. Our approach of integrating computational structural modelling with conventional GWAS analyses and DCA-driven mapping of gene interaction networks has revealed key determinants of *M. abscessus* antibiotic resistance and virulence.

We have discovered three phenotypic clusters, independent of colony morphotype and subspecies, with distinct virulence characteristics and clinical outcomes (not attributable to the known influence of macrolide resistance), that could represent distinct evolutionary trajectories or different points on a single patho-adaptive journey.

To gain systems-level understanding of *M. abscessus* pathobiology, we deployed GWAS analysis, informed by proteome-wide computational structural modelling, to a wide spectrum of in vivo, in vitro and clinical traits, confirming known genetic associations for antibiotic resistance and discovering a large number of unknown genotype–phenotype associations, several of which we validated experimentally. For example, we identified *MbtD*, a polyketide synthase involved in mycobactin synthesis, that regulates intracellular survival of *M. abscessus* and therefore could be targeted therapeutically.

We successfully explored potential epistatic interactions by applying DCA to discover co-evolved proteins and thus inferring networks of potentially functionally linked genes. We confirmed the ability of DCA to reveal gene–gene interactions by comparing outputs with orthogonally derived gene networks created from prior knowledge by the STRING database and experimentally validated the functional relatedness of some of the DCA networks by evaluating CRISPR knockdown of linked genes in both in vitro and in vivo infection assays.

Combining these approaches, we were able to discover several clinically relevant mycobacterial virulence factors. For example, by using a *Drosophila* infection model and structure-guided genomic mapping, we revealed two genes, a putative secretion system protein (*MAB_0471*) and a non-ribosomal peptide synthetase (*MAB_3317c*), that were linked within a DCA-discovered functional network. We validated both genes experimentally and found that both were associated with clinical outcomes in patients.

Our approach capturing and mapping multidimensional phenotypes to genotypes using structural-guided GWAS and defining epistatic interactions through mutational co-evolution can identify clinical relevant phenotypes, virulence-associated mutations and important pathobiological pathways that could be readily applicable to any pathogen, permitting rapid identification of prognostic indicators and potential drug targets.

## Methods

### Sample collection

Samples were obtained from patients with chronic pulmonary disease and respiratory *M. abscessus* infection (baseline characteristics are given in Supplementary Table 1)^3,5^. Isolates were collected in the United Kingdom (all major cystic fibrosis centres), Republic of Ireland (St. Vincent’s Hospital Dublin), United States (University of North Carolina Chapel Hill), Sweden (Gothenborg), Denmark (Copenhagen and Skejby), Australia (Queensland) and the Netherlands (Nijmegen). Where possible, *M. abscessus* samples were obtained from the original mycobacterial growth indicator tubes or from subcultured isolates.

### DNA extraction and whole-genome sequencing

*M. abscessus* cultures were subcultured on solid media and sweeps of multiple colonies collected for sequencing^3,5^. DNA was extracted with the Qiagen QIAamp DNA mini kit. DNA libraries were constructed in pools with unique identifiers for each isolate. Multiplexed paired-end sequencing was performed on the Illumina HiSeq platform. Detailed information on variant calling is provided in the Supporting Information.

### Analysis of bacterial growth on different media

Single *M. abscessus* colonies were picked for phenotypic analysis. Bacterial growth in nutrient-rich medium (Middlebrook 7H9 supplemented with 0.4% glycerol and 10% albumin dextrose catalase enrichment) or carbon source limited media (Middlebrook 7H9 plus carbon source) was assessed in 96-well plates and quantified by measuring the optical density at 600 nm (OD_600_) every 12 or 24 h for 10 d. An OD_600_ above 0.15 assessed in 96-well plates correlated well with log(colony-forming units) (c.f.u.; initial *R*^2^, 0.96; *R*^2^ after 1 d mycobacterial growth in plates, 0.97). The carbon sources tested were acetate (10 mM), glucose (2.5 mM), lactate (10 mM) and pyruvate (10 mM). Growth of each isolate across all conditions was assessed in quadruplicate. For each well, a logistic function was fitted using the R package growthcurver^44^. OD values on day (d)1 were used for early growth and the area under the logistic curve for up to d10 were used to assess general growth. The median of the quadruplicates was used as the representative phenotype. If the readout was highly variable (coefficient of variation >20%) the measurement was considered missing. For assessing potential growth differences of *M. abscessus* mutants, mutants were grown in glass tubes in Middlebrook 7H9 supplemented with 0.4% glycerol and 10% ADC, and assessed daily with a McFarland reader. CRISPRi mutants were additionally supplemented with 100 ng ml^−1^ anhydrotetracycline.

### Drug resistance

Drug resistance was quantified with minimal inhibitory concentrations (MIC) according to the Clinical and Laboratory Standards Institute guidelines^45^. In brief, ~5 × 10^4^ c.f.u. of each isolate were inoculated in increasing antibiotic concentrations in Mueller Hinton broth (amikacin, cefoxitin, clarithromycin and linezolid) or Middlebrook 7H9 supplemented with 0.4% glycerol and 10% ADC (clofazimine) per well. Experiments, including a growth control, were carried out in duplicate for every isolate. The reference strain ATCC 19977 was evaluated once per experimental batch. The MIC was recorded as the lowest drug concentration inhibiting visible growth at d3, d5, d11 and d14. The mean of both experiments (that is, the antibiotic concentration), was recorded and log_2_ transformed. Experiments in which a single MIC could not be obtained (for example, because of visible growth at higher drug concentrations) were excluded.

### Transformation of clinical isolates

An expression plasmid carrying tdTomato (obtained from L. Kremer) was used to transform clinical isolates, grown in 10 ml of Middlebrook 7H9 supplemented with 0.4% glycerol, 10% ADC and 0.05% Tween 80 at 37°C in a shaking incubator. Competent log-phase bacteria were washed with 10% glycerol containing 0.05% Tween 80. Then 200 μl of the pellet together with 1 μg of DNA was transferred to a cuvette and electroporated (2,500 V, 1,000 Ω, 25 μF). Transformed bacteria were recovered for 24 h in antibiotic-free medium and then transferred to a selective agar plate (7H11 complemented with 10% oleic albumin dextrose catalase enrichment and 1 mg ml^−1^ hygromycin). Red colonies were picked and cultured in media containing 1 mg ml^−1^ hygromycin.

### Generation of single cell suspensions

The isolates were obtained from frozen stocks and grown in Middlebrook 7H9 (supplemented with 0.4% glycerol, 10% OADC and 0.05% Tween 80). Exponentially growing isolates were centrifuged at 200*g* for 5 min and the supernatant passed multiple times through a 27-gauge needle before filtrating with a 5 μm filter (Acrodisc syringe filter). Single cell suspensions were standardized to a McFarland turbidity of 0.5 and frozen at −80°C.

### Macrophage infection

THP-1 cells (ATCC TIB-202) were maintained in RPMI 1640 medium supplemented with 10% FCS, penicillin (100 U ml^−1^) and streptomycin (100 U ml^−1^). For infection experiments with clinical *M. abscessus* isolates, around 1 × 10^4^ THP-1 cells per well were differentiated with 20 nM phorbol 12-myristate 13-acetate at 37°C in 384-well imaging plates (CellCarrier-384 Ultra, Perkin Elmer). After 2 d, the adherent, differentiated THP-1 cells were washed and incubated with DMEM supplemented with 10% FCS. On d3 post differentiation THP-1-derived macrophages were inoculated with single cell suspensions of clinical *M. abscessus* isolates at a multiplicity of infection of 1:5, centrifuged for 10 min at 200*g* and incubated at 37°C. After 2 h extracellular cells were washed off. After 2, 24 or 48 h cells were stained with CellMask DR (Invitrogen) for 20 min, washed, fixed with 4% paraformaldehyde for 1 h and stained with 4,6-diamidino-2-phenylindole. The cell supernatant was stored at −80°C. The macrophage infection experiments of 245 tdTomato-expressing clinical isolates were set up in quadruplicate at once for all time points (2, 24 and 48 h). THP-1 infection experiments with *M. abscessus* mutants were carried out similarly, with the exception that they were done in 96-well plates with around 1 × 10^5^ THP-1 cells per well, and in case of CRISPRi mutants supplemented with 100 ng ml^−1^ anhydrotetracycline, starting 24 h before infection. After 2, 24 and 48 h, cells were washed three times, lysed with H_2_O and the number of c.f.u. was assessed. In total, three CRISPRi mutants were generated per gene, assessed in triplicate and analysed per gene.

### High-content image acquisition and analysis

After paraformaldehyde fixation plates were stored at 4°C and imaged within 24 h on the high-content screening platform Opera Phenix (Perkin Elmer). Spinning disc confocal images of 37 fields per well and three fluorescence channels (blue 405/456, red 561/599, far-red 640/706) were acquired with a ×63 water immersion objective (NA 1.15). Automated image analysis was performed with Columbus software (v.2.9.0, Perkin Elmer). The 37 fields were pooled to single wells. Blue (4,6-diamidino-2-phenylindole) and far-red (CellMask DR) fluorescence channels were used to define cells and their borders. To evaluate the viability of individual macrophages, a supervised machine learning approach (Columbus; Perkin Elmer) based on nuclear, cytosolic and cell features was used to train a linear classifier, which was then applied to all images to classify macrophages as dead or alive. Intra- and extracellular mycobacteria were defined using a spot assay on the red fluorescence channel. For each cell, as well as the extracellular space, the spot area and mean fluorescence intensity were documented. Both measures were used to quantify the mycobacterial load (intracellular load = total sum of (spot area per cell × mean spot intensity per cell); extracellular load = extracellular spot area × extracellular mean spot intensity; total mycobacterial load = intracellular load + extracellular load). Wells with a cell number below 800 were removed; the median of the remaining wells was used. As the most meaningful outputs we reported the fraction of total cells infected (number of *M. abscessus* infected cells/total number of cells), the intracellular and total *M. abscessus* load as well as the fraction of cells alive (number of cells alive/total number of cells). Mycobacterial load or cell kinetics are reflected in the ratio d2/d0 (delta).

### Cytokine assessment

The supernatant of macrophages was evaluated for interleukin-8 and tumour necrosis factor-α concentrations 24 h after mycobacterial infection. Tumour necrosis factor-α and interleukin-8 levels were measured in 25 µl of supernatant on a Luminex 200 instrument (Merck Millipore) using the reagents and protocol supplied with the Milliplex MAP Human Cytokine/Chemokine kit (Merck Millipore).

### *Drosophila* infection

Isogenic flies (w^1118^) were maintained using standard fly medium (2% polenta, 10% Brewer’s yeast, 0.8% agar, 8% fructose and water) at 25°C. Flies were infected with inducible CRISPRi mutants of *M. abscessus* and put on fly medium supplemented with tetracycline (0.2 mg ml^−1^) several days before infection. Details on fly infection procedures are provided in the Supporting Information. Some 400 c.f.u. were injected in 50 nl of PBS into the abdomen of anaesthetized 6–8-d-old male flies. Around 15 flies per condition (in total >350 conditions) were infected to assess survival. Fly survival was assessed every 12 h until d10 and compared using the log-rank test.

### Quantitative PCR with reverse transcription of *Drosophila* antimicrobial peptides and cytokines

At least five flies were infected with each isolate to assess the immune response to infection. At 28 h after infection, flies were homogenized in 100 μl of TRIzol (Invitrogen) and stored at −20°C. RNA was then extracted and complementary DNA synthesis was carried out with the RevertAid Reverse Transcriptase (200 U µl^−1^, Thermo Fisher Scientific). Quantitative PCR analyses were performed in duplicate using the Sensimix SYBR no-ROX kit (Bioline)^46,47^ using the primers given in Supplementary Table 2.

### Patient outcomes

Clinical outcome data were available for 300 CF patients (as reported previously^3,5^). Patients were classified as having cleared *M. abscessus* infection (defined as documented culture conversion or a sustained clinical improvement where further cultures were unavailable) or as having persistent infection (if cultures remained positive or the clinical state worsened where no cultures were available)^5^. Lung function decline was estimated as the percentage change in the forced expiratory volume from the available lung function assessment over a period of 12 months from baseline (before infection).

### Phenotype association

To assess relatedness of phenotypes and phenotypic groups, all phenotype pairs were correlated (Pearson correlation) and a correlation matrix plotted. To identify characteristic phenotypic signatures of clinical isolates, isolates were clustered using representative experimental phenotypes (amikacin MIC d11, clarithromycin MIC d11, growth d10, change in intracellular MAB load, macrophage cell death d2, *Drosophila* attacin level, mean *Drosophila* survival). Some 199 isolates with at most one missing value (52 isolates had one missing value) were correlated using pairwise Pearson correlation. The resulting correlation matrix was used as a distance measure to cluster isolates with *t*-SNE^48^ using the R package Rtsne. Clustering was validated with *k*-means clustering with a predefined set of three clusters. Phenotypic groups were compared using one-way analysis of variance or chi-squared test, as appropriate, and mapped onto the phylogeny. For each isolate a nearest phylogenetic neighbour was identified, thereby assessing whether neighbours are more likely to belong to the identical phenotypic group (chi-squared of each phenotypic group comparing neighbour pairs versus non-neighbour pairs).

### Genome-wide association analysis

Two statistical genome-wide association approaches were employed to assess the effect of individual variants (SNPs, INDELs, large deletions) on phenotypes. A linear mixed model controlling for population structure, where the phenotype is modelled on the fixed locus effect and the random effect of the relatedness matrix, was used. However, controlling for population structure considerably reduces power for population-stratified variants^23^. Because population-stratified variants are common in bacteria, genome-wide associations were also analysed with a linear model. Both analyses were performed in GEMMA^22^. Hits were defined as the top 50 significant associations within a phenotype. Manhattan plots were generated using LocusZoom^49^.

### Genome-wide protein structure prediction

Because the structures of most proteins in the *M. abscessus* proteome have not been resolved experimentally, it was necessary to model them computationally. We therefore extended our *M. abscessus* structural proteome database, Mabellini^29^, which provides only high-confidence, well-annotated structural data, to aim for comprehensive coverage of the entire proteome. Therefore, additional proteins were modelled with lower-confidence templates aided with extensive macromolecular modelling and refinement protocols. The multiple sequence alignments were converted into profile hidden Markov models (HMMs) using HH-suite3 (ref. ^50^), which were then used to search against a pdb70 (Protein Data Bank chains clustered at 70% sequence identity) database using Hhsearch^50^. The identified templates were used for comparative modelling, using the modified, MODELLER-based^51^, multi-template structure modelling pipeline of Larsson et al.^52^. In addition to structural consensus and a machine learning-based single-model quality assessment protocol, we also incorporated a rapid method for annotating the quality of protein models through comparison of their distance matrices^53^. As a result, for each of the modelled protein sequences, we obtained a set of theoretical models, ranked by predicted model quality.

### Machine learning for assessing effects of missense mutations

To evaluate the effect of polymorphisms on *M. abscessus* protein structures, we used the models generated in the previous step to estimate the effect of missense mutations. We applied mCSM^28^, which, through graph-based signatures, represents the structural environment of wild-type residues and learns which mutations are detrimental to protein structure. For each of the mutations, one or more modelled structures have been used.

### Comparative modelling of *MAB_2119c* (*MbtD*)

The model of putative polyketide synthase (mbtD, MAB_2119c) was produced as part of Mabellini using the following models: 2hg4, 3tzz and 2jgp^29^. The Mabellini-derived structure was then subjected to extensive relaxation using Rosetta^54^ suite, in both a wild-type and mutated variants, where the lowest energy structure has been chosen for subsequent analysis.

### Ranking of predicted functional impact of SNPs

Based on SNP annotation (intergenic, synonymous, inframe INDEL, frameshift) and structural modelling predictions of functional impact (above), variants were allocated to four groups: low-effect variants (intergenic and synonymous SNPs; grey), low–moderate-effect variants (inframe INDEL, missense mutations with lowest tertile mCSM scores; green), moderate–high-effect variants (missense mutations with middle tertile mCSM scores; blue) and high-effect variants (frameshift variant, large deletion, start/stop alteration and missense mutations with highest tertile mCSM scores; red).

### Summary of GWAS hits

To summarize the identified variants across all phenotypes, up to five significant, highest ranking hits were extracted from each genotype–phenotype association (a single high- or moderate-effect variant per gene). In total, 2 × 58 genotype–phenotype associations (linear mixed model and linear model) were performed. To assess genetic linkage between these variant hits, we calculated *R*^2^ using PLINK^55^.

### Identification of homologues and construction of multiple sequence alignments

For each of the proteins in the *M. abscessus* proteome, we have constructed a multiple sequence alignment of homologous proteins, which forms a basis for subsequent work. The alignments have been constructed using HHblits, a fast, highly sensitive, HMM–HMM-based sequence search method^56^ and used the bundled nr30 database. In the interest of exploring a broader evolutionary landscape of proteins in question, we have decided to include proteins with an E-value ≤10^−4^ in the alignment.

### Genome-wide evolutionary coupling inference

Exponential models to understand co-evolution in biological sequences have been applied to protein structure prediction^57^, and more recently to bacterial genomic sequences. We have previously shown that the method genomeDCA^33^ can be effectively employed to understand the co-evolution of *Streptococcus pneumoniae*^34^, and is extensible and applicable to other systems^32,34,58^. Here, we employ an approach that blends genomeDCA^33^ and CC-DCA^32^ to ensure unbiased sampling of evolutionary pressures onto individual positions and pairs of positions across genomic sequences. CC-DCA^32^ permits genome-wide coupling inference without needing to resort to extensive sampling, as proposed in genomeDCA^33^. We modified this approach to elucidate the effects of low-frequency alleles across the entire *M. abscessus* genome. We conducted at least 60,000 runs, each subsampling 25% of positions in the genome. We defined variant–variant couplings as statistically significant based on the Gumbel distribution (as described previously^33^) corresponding to an FDR of <10^−6^. Variant–variant pairs that spanned a distance of more than 100 bp were ranked by coupling strength and visualized on the *M. abscessus* genome using the Circos package^59^. Subsequently, we pooled the statistically significant couplings by gene–gene pairs, and ranked them by the number of couplings. Cytoscape was used to plot the network of the 1,000 strongest gene–gene couplings, highlighting the number of couplings (edge width), coupling strength (edge colour) and predicted gene function (node colour)^60^. For CC-DCA validation, we assessed the protein–protein interactions of putative functional clusters with STRING v.11.5 (nodes, observed and expected edges, protein-protein interaction enrichment *P* value)^36^.

### Generation of CRISPRi mutants

Analogous to CRISPR-mediated gene silencing in *Mycobacterium tuberculosis* and *Mycobacterium smegmatis*, we established a CRISPRi platform in *M. abscessus*^35,43,61^*. M. abscessus* ATCC 19977 was transformed with pTetInt-dCas9 and a second vector (pGRNAz) containing the small-guide RNA cassette. For each gene, two oligonucleotides were synthesized (forward and reverse), annealed and cloned into pGRNAz. Oligonucleotide sequences are outlined in Supplementary Table 3. The strains were grown in Middlebrook 7H9 broth (supplemented with 0.4% glycerol, 10% ADC and 0.05% Tween 80) and selected with hygromycin (1 mg ml^−1^) and zeocin (300 μl ml^−1^). dCas9 and sgRNA expression were under the control of a tet-inducible promotor. To achieve maximal gene repression cultures were supplemented with 100 ng ml^−1^ anhydrotetracycline. As controls, an empty vector control and YidC (essential gene) knockdown were used. To validate CRISPR-induced transcriptional repression we complemented knockdown mutants with rescue vectors, in which *MAB_0471* or *MAB_472* containing silent mutations at the CRISPR-binding sites were cloned into pGRNAz under a strong promoter. In these mutants, CRISPR guides bind and repress chromosomal gene expression, but not the mutated gene expressed in the plasmid.

### Generation of knockout and complemented mutants

To validate structural predictions, a *MbtD* knockout mutant was generated on the ATCC 19977 background via recombineering^62^. In brief, primers which amplified the 1,000-bp flanking regions up- and downstream of the respective gene were designed and a zeocin cassette was cloned between these fragments to synthetize an allelic exchange substrate. pJV53 was used to generate the recombineering strain ATCC19977-pJV53, which was grown to the exponential phase and induced with 0.2% acetamide^44^. The allelic exchange substrate was then electroporated into ATCC19977-pJV53 and plated on Middlebrook 7H11 agar supplemented with 10% OADC containing 300 μg ml^−1^ zeocin and then grown in broth culture to remove pJV53. To complement ΔMAB_2119, MAB_2119 was PCR-amplified, digested and ligated into pMV306-hsp60. To generate ΔMAB_2119 + Ile256Thr and ΔMAB_2119 + Thr410Ala complemented mutants, pMV306-MAB_2119 was PCR-amplified using oligonucleotides containing the chosen mutation (Supplementary Table 3). These plasmids were then electroporated into ΔMAB_2119 on Middlebrook 7H11 agar supplemented with 10% OADC and kanamycin (200 μg ml^−1^) and confirmed by PCR.

### Ethics approval

Ethical approval was obtained from the National Research Ethics Service (NRES; REC reference: 12/EE/0158) and the National Information Governance Board (NIGB; ECC 3-03 (f)/2012) for centres in England and Wales; from NHS Scotland Multiple Board Caldicott Guardian Approval (NHS Tayside AR/SW) for Scottish centres; and respective review boards from Queensland (Australia) and the University of North Carolina (USA).

### Reporting summary

Further information on research design is available in the Nature Research Reporting Summary linked to this article.

## Supplementary information

Supplementary Information Supplementary methods, figure legends, figures, references and Tables 1–4.

Reporting Summary

Supplementary Data 1 GWAS hits across all phenotypes.

Supplementary Data 2 1000 strongest gene–gene couplings.

Supplementary Data 3 Sequencing accession numbers.

Supplementary Data 4 Clarithromycin resistance and rrl and erm(41) genotypes.

Supplementary Data 5 SDS Fig 8B: *Drosophila* survival with virulence variant. SDS Fig 8 C: Outcome with virulence variant. SDS Fig 8D: Sampling after NTM onset across virulence variants. SDS Fig 8E: MAB_0472 gene expression in *Drosophila* in control and MAB_0472 knockdown mutants. SDS Fig 8F: *Drosophila* survival in MAB_0471 knockdown and complemented mutants. SDS Fig 8G: *Drosophila* survival in MAB_0472 knockdown and complemented mutants.

Supplementary Data 6 Drosophila survival of MAB mutants.

Supplementary Data 7 SDS Fig 10A: Coverage depth of 330 MAB isolates. SDS Fig 10B: Coverage frequency of 20 bp windows. SDS Fig 10D: Large deletions across the MAB genome. SDS Fig 10E: *Drosophila* survival with different inocula in different isolates. SDS Fig 10F: Mean *Drosophila* survival with different inocula in different isolates.

## Data Availability

All sequencing data of this study is deposited in the European Nucleotide Archive with the respective accession codes provided in Supplementary Data. Source data are provided with this paper.

## Source data

Source Data Fig. 1 Phenotypic data.
Source Data Fig. 2 Phenotypic data, outcome data, morphotypes, subspecies and phenotypic groups.
Source Data Fig. 3 Fig. 3a: GWAS summary linear and mixed model, linkage disequilibrium. Fig. 3d: Growth and intracellular MAB change in control and MAB *MbtD* mutants.
Source Data Fig. 4 Fig. 4a: Variant–variant couplings. Fig. 4b: Gene–gene and variant–variant couplings per gene. Fig. 4c,d: Gene–gene couplings. Fig. 4e: Change of intracellular MAB count in mutants of the mycobactin cluster.
Source Data Fig. 5 Fig. 5c: *Drosophila* survival and virulence variants. Fig. 5d: *Drosophila* survival and outcomes in virulence variant. Fig. 5e: Growth and *Drosophila* survival in control and mutant strains (virulence genes). Fig. 5g: *Drosophila* survival in interacting in control and mutant strains (interacting gene).

## Code availability

All code used in this study has been previously published.
