# Supplementary material for: Mycobacterium abscessus pathogenesis identified by phenogenomic analyses
Source: Nat Microbiol. 2022 Aug 25;7(9):1431–41. doi: 10.1038/s41564-022-01204-x (PMC9418003; doi:10.1038/s41564-022-01204-x)
Supplement: Supplementary file 1 — Supplementary methods, figure legends, figures, references and Tables 1–4. [file 41564_2022_1204_MOESM1_ESM.pdf]

nature portfolio

https://doi.org/10.1038/s41564-022-01204-x

Supplementary information

Mycobacterium abscessus pathogenesis identified by phenogenomic analyses

In the format provided by the
authors and unedited

Mycobacterium abscessus pathogenesis identified by phenogenomic analyses

Lucas Boeck[1,2,3,4], Sophie Burbaud[1,2], Marcin Skwark[5], Will H. Pearson[6,7], Jasper Sangen[1,2], Andreas W. Wuest[4], Eleanor K. P. Marshall[6,7], Aaron Weimann[1,2], Isobel Everall[3], Josephine M Bryant[1,2], Sony Malhotra[5,8], Bridget P. Bannerman[1,2,5], Katrin Kierdorf[6,7,9], Tom L. Blundell[5], Marc S. Dionne[6,7], Julian Parkhill[10], R. Andres Floto[1,2,11]

SUPPLEMENTARY METHODS

Variant calling

The quality of each sample, was assessed on *de novo* assemblies. Assemblies longer than 6Mb, with more than 300 contigs, an average depth of coverage below 30x, a coverage of the reference genome below 50% or evidence of a mixed infection were discarded. Sequence reads, from 2366 samples, were mapped with BWA to the *Mycobacterium abscessus* reference genome (ATCC19977) followed by an INDEL realignment step using GATK (total alignment) [1–3]. Furthermore, a single random sequence per patient was picked to generate an alignment with a single sample per patient (single patient alignment). In total, 484 clinical isolates plus the ATCC19977 strain were included in the single patient alignment. Bcftools was used for SNP and small INDEL calling where additional criteria were used to filter SNPs, requiring a minimum base call quality of 50, a minimum mapping quality of 20 and a minimum number of matching reads covering a SNP of 8 (3 per strand) [4]. SNPs were annotated with SNPeff [5]. In addition, we assessed larger deletions, which commonly drive phenotypic diversity in mycobacteria [6]. However, with common approaches it remains difficult to resolve complex rearrangements and predicting its genomic coordinates can sometimes be impossible. Since the coverage of our *M. abscessus* whole genome sequences was moderate to high (overall mean coverage: 75x, ***Supplementary Figure 10***) we explored if we could use a very low coverage for identifying larger deletions at specific genomic coordinates. The *M. abscessus* reference genome was partitioned into regions of 20bp with 10bp overlaps and the coverage of these regions in clinical isolates assessed with sambamba [7]. While most 20bp windows had a coverage between 50-150x, we observed only very few windows with a coverage of 10-25x. Large deletions did neither have a higher GC content, nor were they enriched in GCC/CCG triplets [8]. Windows with a mean coverage of 5x or lower, with a minor allele frequency across all genomes greater than 5% and occurring in at least two consecutive windows were called as large deletions. If the distribution of consecutive deletions was equal across all isolates these variants were collapsed into a single variant. Using this method we observed 23.426 regions with large deletions across 871 genes, but only in 9 essential genes, ranging from 0 (closest neighbour of ATCC19977) up to 20.936 large deletions in single

isolates. A maximum likelihood tree of 331 samples assessed for *Drosophila* survival, inferred from SNPs was constructed with FastTree [9] and plotted using itol [10].

Assessment of *M. abscessus* morphotypes

The clinical isolates were plated on agar plates (Middlebrook 7H11 agar supplemented with 10% OADC) and their morphotypes, smooth or rough, macroscopically assessed between day 3 and day 5. All isolates were plated and assessed twice on different days. Isolates with a consistent rough morphotype were considered rough.

***Drosophila* infection**

For *Drosophila* infections[11–14], a pulled glass capillary needle was attached to a microinjection dispense system (Picospritzer) and the solution containing a single cell suspension of *M. abscessus* aspirated into a glass capillary [11–14]. The microinjection system was then individually calibrated by visually assessing and adjusting the droplet size to 50nl. In order to increase reproducibility and dynamic range across different clinical isolates we optimised the *M. abscessus* inoculum (***Supplementary Figure 10***). 400 CFUs were injected in 50nl PBS into the abdomen of anaesthetised 6-8 day old male flies. Flies were kept on CO_2 for a maximum of 10min, transferred to a new vial and kept at 29°C. The sample size was chosen to provide accurate and statistically comparable measures of *Drosophila* survival. Around 15 flies per condition (in total >350 conditions) were infected. Fly survival was assessed every 12h until day 10. Due to bio-safety concerns no additional fly food was provided after infection reducing survival in control flies to a mean survival of about 8 days. In order to minimise technical effects related to fly infection, the mean fly survival was calculated excluding the flies dying within the first 3 days and flies where death deviated more than 3 SD from the mean. Fly survival was compared using the log rank test.

GWAS threshold

The GWAS threshold, i.e. correction for multiple hypothesis testing, was calculated on the effective number of independent high and moderate effect variants. Within the 331 isolates phenotyped for *Drosophila* survival we obtained in total 75260 high/moderate effect variants (large deletions, frameshifts, start/stop alterations, missense mutations) with a minor allele frequency above 0.03. The assumption of Bonferroni correction is that individual tests are independent, which is very conservative when considering genetic variants in extensive linkage disequilibrium (LD), impairing statistical power. In order to correct for variant dependency due to LD we assessed the Bonferroni threshold on the effective number of independent tests [15]. Using the genetic type 1 error calculator (GEC) on 75260 genotypes we revealed 17925 independent tests, which were used to calculate a Bonferroni corrected

threshold ($2.8*10^{-6}$) [24]. This threshold was applied for all genome-wide associations studies, including those with less variants.

Identification of homologs and construction of multiple sequence alignments

For each of the proteins in the *M. abscessus* proteome, we have constructed a multiple sequence alignment of homologous proteins, which formed a basis for subsequent work. The alignments have been constructed using HHblits, a fast, highly sensitive, HMM-HMM-based sequence search method [16] and used the bundled nr30 database. In the interest of exploring a broader evolutionary landscape of proteins in question, we have decided to include proteins with E-value less than or equal to 10^{-4} in the alignment.

qRT-PCR of mycobacterial genes within *Drosophila*

qRT-PCRs to validate CRISPR-induced mycobacterial knockdown of *MAB_472* within the *Drosophila* infection model was carried out similarly to qRT-PCRs of antimicrobial peptides. Four days post infection, three flies per sample were homogenized in TRIzol, transferred into a 2 mL tube containing 500 uL of zirconium beads and bead beated twice for 2 minutes before RNA extraction using chloroform and isopropanol. Following a DNAse treatment cDNA was synthetised using Revertaid M-MuLV reverse transcriptase and random hexamers. qPCRBIO Probe Mix was used for TaqMan qRT-PCR. Primers and FAM-TAMRA taqman probes used are listed in *Supplementary Table 3*. The gene expression of each sample was calculated based on the standard curve produced and normalised to the cDNA concentration of the sample. At least 5 biological replicates were used per group.

SUPPLEMENTARY FIGURE LEGENDS

Supplementary Figure 1: Distribution of *in vitro* phenotypes and clinical outcomes across *M. abscessus* isolates. (**a**) Planktonic growth of clinical *M. abscessus* isolates in five different carbon sources was assessed after one day (d1) and 10 days (d10). (**b**) Minimal inhibitory concentrations of five different antibiotics measured on several days. (**c**) Distribution of clinical outcomes in *M. abscessus* lung disease (clearance vs persistent infection and lung function decline), associated with *M. abscessus* isolates.

Supplementary Figure 2: Distribution of macrophage and *in vivo* infection across *M. abscessus* isolates. (**a**) Phenotypes associated with THP-1 macrophage infection, such as the proportion of macrophages infected, *M. abscessus* intracellular replication and macrophage death (all assessed via high-content imaging) and cytokine release at 24h post infection (**b**) *Drosophila* survival and antimicrobial peptide expression after *in vivo M. abscessus* infection.

Supplementary Figure 3: Characteristics of phenotypic clusters and macrolide resistance. (**a**) Pearson correlation matrix of *in-vitro* and *in-vivo* bacterial phenotypes and clinical outcomes (all phenotypes labelled and correlations annotated). (**b**) The 7 phenotypes bacterial growth, drug resistance (amikacin and clarithromycin), intracellular replication, macrophage death, Drosophila survival and antimicrobial response were used to group clinical *M. abscessus* isolates. The distribution of specific phenotypes across these three revealed clusters are illustrated and compared using a two-sided one-way analysis of variance (Bacterial growth: p=3.6e-7; intracellular bacterial replication: p=1.8e-10; macrophage death: p=5.1e-10; *Drosophila* survival: p=8.5e-11; clarithromycin resistance: p=2.5e-115). (**c**) Maximum likelihood phylogenetic tree of 294 *M. abscessus* isolates, annotated for macrolide resistance phenotypes (clarithromycin MIC day 11) and genotypes conferring acquired and inducible macrolide resistance.

Supplementary Figure 4: Experimental and clinical phenotypes across *M. abscessus* morphotypes and subspecies. (**a**) Phenotype distribution in smooth and rough clinical isolates (two-sided unpaired t test). (**b**) Clinical outcomes do not differ across *M. abscessus* morphotypes (two-sided unpaired t test or Fisher's exact test). **(c**) Phenotypic clustering reveals similar phenotypic groups when only isolates with a smooth morphotype are used. (**d**) *M. a. massiliense* isolates are generally more susceptible to macrolides (p=1.0e-12). Other phenotypes are broadly similar across subspecies (two-sided one-way analysis of variance). (**e**) Clinical outcomes do not differ between subspecies. (**f**) Phenotypic clustering reveals

similar phenotypic groups when only *M. a. abscessus* isolates are used (two-sided one-way analysis of variance or Chi-squared test).

Supplementary Figure 5: Genome-wide association of known resistance mechanisms. Genotype-phenotype associations of (**a**) amikacin and (**b**) clarithromycin MICs day 3 revealed the known resistance loci in the 16S and 23S ribosomal RNA, respectively. (**c**) *Erm(41)* conferring inducible macrolide resistance, was identified when assessing genetic associations with clarithromycin MICs at day 11 in *M. a. abscessus*. Linear and mixed models were used and p values calculated with the Wald test. The black horizontal lines mark the multiple hypothesis testing threshold based on the number of independent variants.

Supplementary Figure 6: Linkage disequilibrium and co-evolving variant couplings. (**a**) Analysis of linkage disequilibrium of *M. abscessus* variants. Pairwise R^2 measurements of variants (as shown in Figure 3) now ordered by genomic position. (**b**) Distribution and threshold of the variant-variant coupling strength in *M. abscessus*. Divergence of theoretical (fitted distribution) and empirical distribution of the coupling strength. The dashed line highlights the defined threshold (coupling strength above 0.080) with a false discovery rate of 1 in 10^6 couplings.

Supplementary Figure 7: Comparison of gene networks generated by Direct Coupling Analysis and *STRING*. Gene networks generated from ccDCA (from Figure 4; node colour representing gene ontology; edge thickness and shading representing coupling number and strength respectively) are compared to *STRING* network analysis of the same genes (edge colour representing supporting evidence type; metrics provided for network node number, expected and observed edges, and *STRING* calculated protein-protein interaction enrichment).

Supplementary Figure 8: Description and validation of virulence variants. (**a**) Virulence variants around *MAB_0471*. Genetic variants of *M. abscessus* associated with *Drosophila* survival (revealed with Wald test statistics) mapped around *MAB_0471* (described in Figure 5). High-effect genetic variants shown in red, moderate-high in blue, low-moderate in green and low in grey. The black horizontal line marks the multiple hypothesis testing threshold. Clinical outcomes related to missense variant in the peptide synthetase *MAB_3317c* and sampling after NTM onset. The missense mutation K1835E in *MAB_3317* was associated with (**b**) a prolonged mean *Drosophila* survival (two-sided unpaired t-test; p=1.0e-13) and (**c**) a higher fraction of persistent respiratory *M. abscessus* infections in Cystic Fibrosis patients (two-sided Chi-squared test). (**d**) The two variants in *MAB_0471* and *MAB_3317* were not

associated with the time of sampling after onset of NTM lung disease (two-sided unpaired t-test). (**e**) Validation of *GWAS* hits for *Drosophila* survival. Violin plot with median of *MAB_472* expression in control (n=12 biologically independent samples) or knockdown mutants (n=17 biologically independent samples of 3 guides with 5 or 6 replicates each) extracted from infected *Drosophila* (groups were compared using a two-sided unpaired t-test). *Drosophila* survival curves uninfected (*green*), infected with control *M. abscessus (grey), MAB_471* (**f**) or *MAB_472* (**g**) knockdown mutants *(*red), or knockdown mutants complemented with CRISPR-untargetable genes (*black*). *** p<0.0001 (survival compared to the knockdown mutant using a two-sided log rank test; MAB_0471 KD vs. MAB control: p=6.5e-17; MAB_0471 KD vs. MAB_0471 KD::MAB_0471: p=4.2e-17; MAB_0472 KD vs. MAB control: p=4.0e-11; MAB_0472 KD vs. MAB_0472 KD::MAB_0472: p=1.1e-5). (**h**) Epistatic interactions of *MAB_0471* and *MAB_3317*. Gene interaction network generated using ccDCA (shown in Figure 5) now annotated by gene ontology.

Supplementary Figure 9: CRISPR/dCas9 knockdown with different guide RNAs. Kaplan-Meier plots comparing three knockdowns per gene with empty vector controls using the log-rank test (MAB_0471 sgRNA1: p=2.9e-9; MAB_0471 sgRNA2: p=4.5e-5; MAB_0472 sgRNA1: p=1.8e-6; MAB_0472 sgRNA2: p=3.1e-8; MAB_0472 sgRNA3: p=1.6e-9; MAB_3317 sgRNA1: p=4.6e-6; MAB_3317 sgRNA2: p=2.9e-7; MAB_3317 sgRNA3: p=1.4e-10).

Supplementary Figure 10: Rationale for large deletion assessments and *Drosophila* infection inoculum (**a**) Large deletions of the *M. abscessus* genome. Moderate-high coverage of the sequenced *M. abscessus* genomes. (**b**) Gap of 20bp windows with a mean coverage of 10-25x. Two consecutive 20bp windows with a coverage of 5x or below and occurring in at least 5% of all *M. abscessus* isolates were considered a large deletion (**c**) Artemis illustration of a representative large deletion in *MAB_1137c* associated with a rapid loss of coverage (mapped reads), independent of GC content. (**d**) Distribution of large deletions within 871 genes (black) across 330 clinical isolates and ATCC19977. (**e**) *Drosophila* infection inoculum. Different inocula were used to optimise the *Drosophila* infection inoculum (>15 flies per condition) (**f**) The best reproducibility was achieved with 500 colony forming units. The highest dynamic range across different clinical isolates was observed with smaller inocula. Accordingly, we used 400 colony forming units per fly to screen 331 *M. abscessus* isolates using the *Drosophila* infection model.

SUPPLEMENTARY REFERENCES

1. Li, H. & Durbin, R. Fast and accurate long-read alignment with Burrows-Wheeler transform. *Bioinformatics* **26**, 589–595 (2010).

2. McKenna, A. *et al.* The Genome Analysis Toolkit: a MapReduce framework for analyzing next-generation DNA sequencing data. *Genome research* **20**, 1297–1303 (2010).

3. Ripoll, F. *et al.* Non Mycobacterial Virulence Genes in the Genome of the Emerging Pathogen Mycobacterium abscessus. *Plos One* **4**, (2009).

4. Li, H. *et al.* The Sequence Alignment/Map format and SAMtools. *Bioinformatics* **25**, 2078–2079 (2009).

5. Cingolani, P. *et al.* A program for annotating and predicting the effects of single nucleotide polymorphisms, SnpEff: SNPs in the genome of Drosophila melanogaster strain w(1118); iso-2; iso-3. *Fly* **6**, 80–92 (2012).

6. Bottai, D. *et al.* TbD1 deletion as a driver of the evolutionary success of modern epidemic Mycobacterium tuberculosis lineages. *Nat Commun* **11**, 684 (2020).

7. Tarasov, A., Vilella, A. J., Cuppen, E., Nijman, I. J. & Prins, P. Sambamba: fast processing of NGS alignment formats. *Bioinformatics* **31**, 2032–2034 (2015).

8. Carver, T., Harris, S. R., Berriman, M., Parkhill, J. & McQuillan, J. A. Artemis: an integrated platform for visualization and analysis of high-throughput sequence-based experimental data. *Bioinformatics* **28**, 464–469 (2012).

9. Stamatakis, A. RAxML-VI-HPC: maximum likelihood-based phylogenetic analyses with thousands of taxa and mixed models. *Bioinformatics* **22**, 2688–2690 (2006).

10. Letunic, I. & Bork, P. Interactive Tree Of Life (iTOL) v4: recent updates and new developments. *Nucleic Acids Res* **47**, W256–W259 (2019).

11. Dionne, M. S., Ghori, N. & Schneider, D. S. Drosophila melanogaster is a genetically tractable model host for Mycobacterium marinum. *Infection and immunity* **71**, 3540–3550 (2003).

12. Pean, C. B. *et al.* Regulation of phagocyte triglyceride by a STAT-ATG2 pathway controls mycobacterial infection. *Nature Communications* **8**, 1–11 (2017).

13. Oh, C.-T., Moon, C., Jeong, M. S., Kwon, S.-H. & Jang, J. Drosophila melanogaster model for Mycobacterium abscessus infection. *Microbes Infect* **15**, 788–795 (2013).

14. Oh, C.-T., Moon, C., Park, O. K., Kwon, S.-H. & Jang, J. Novel drug combination for Mycobacterium abscessus disease therapy identified in a Drosophila infection model. *J Antimicrob Chemoth* **69**, 1599–1607 (2014).

15. Li, M.-X., Yeung, J. M. Y., Cherny, S. S. & Sham, P. C. Evaluating the effective numbers of independent tests and significant p-value thresholds in commercial genotyping arrays and public imputation reference datasets. *Human genetics* **131**, 747–756 (2012).

16. Remmert, M., Biegert, A., Hauser, A. & Söding, J. HHblits: lightning-fast iterative protein sequence searching by HMM-HMM alignment. *Nature methods* **9**, 173–175 (2011).

Supplementary Figure 1

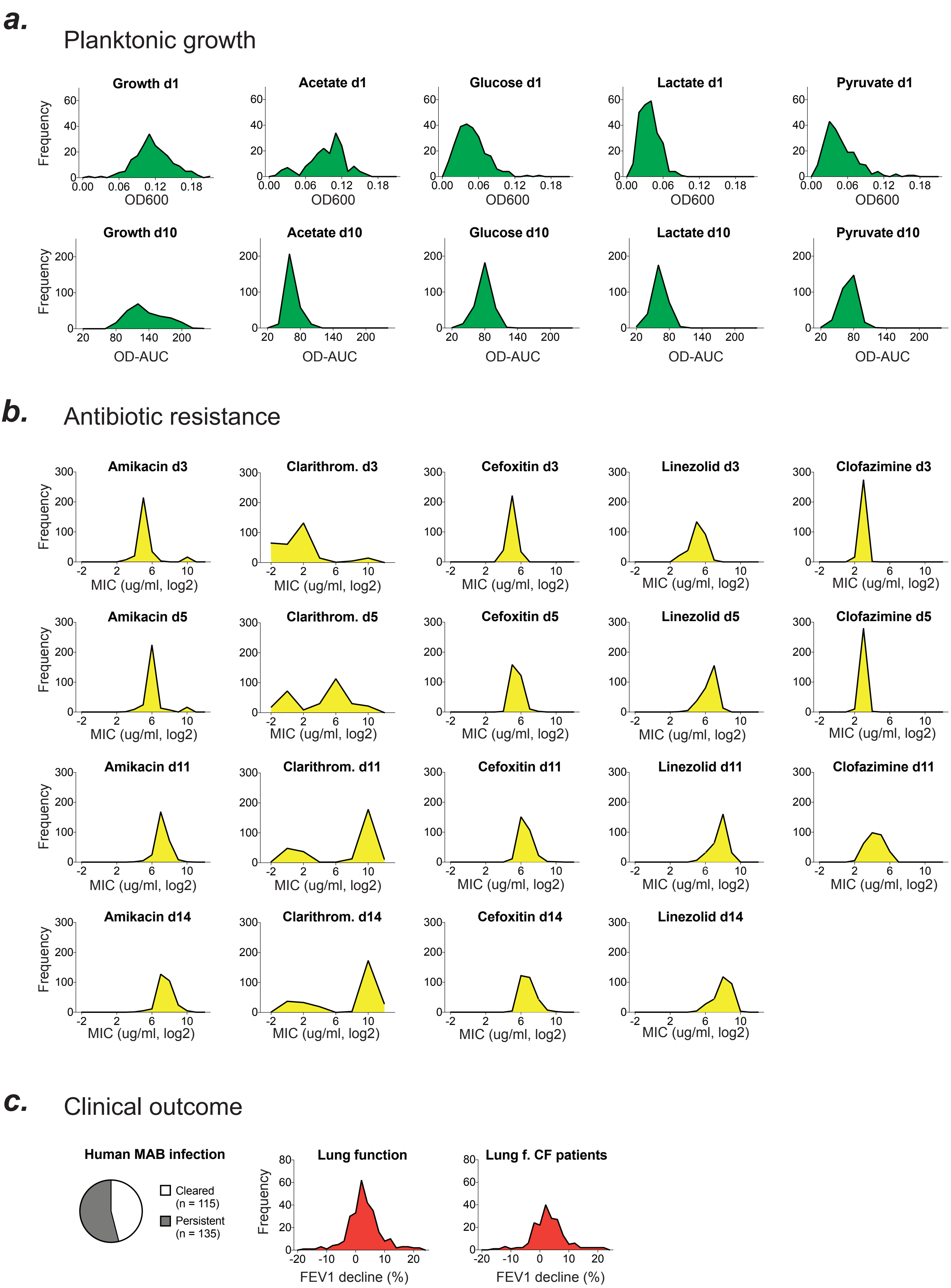

Supplementary Figure 2

a. Macrophage infection

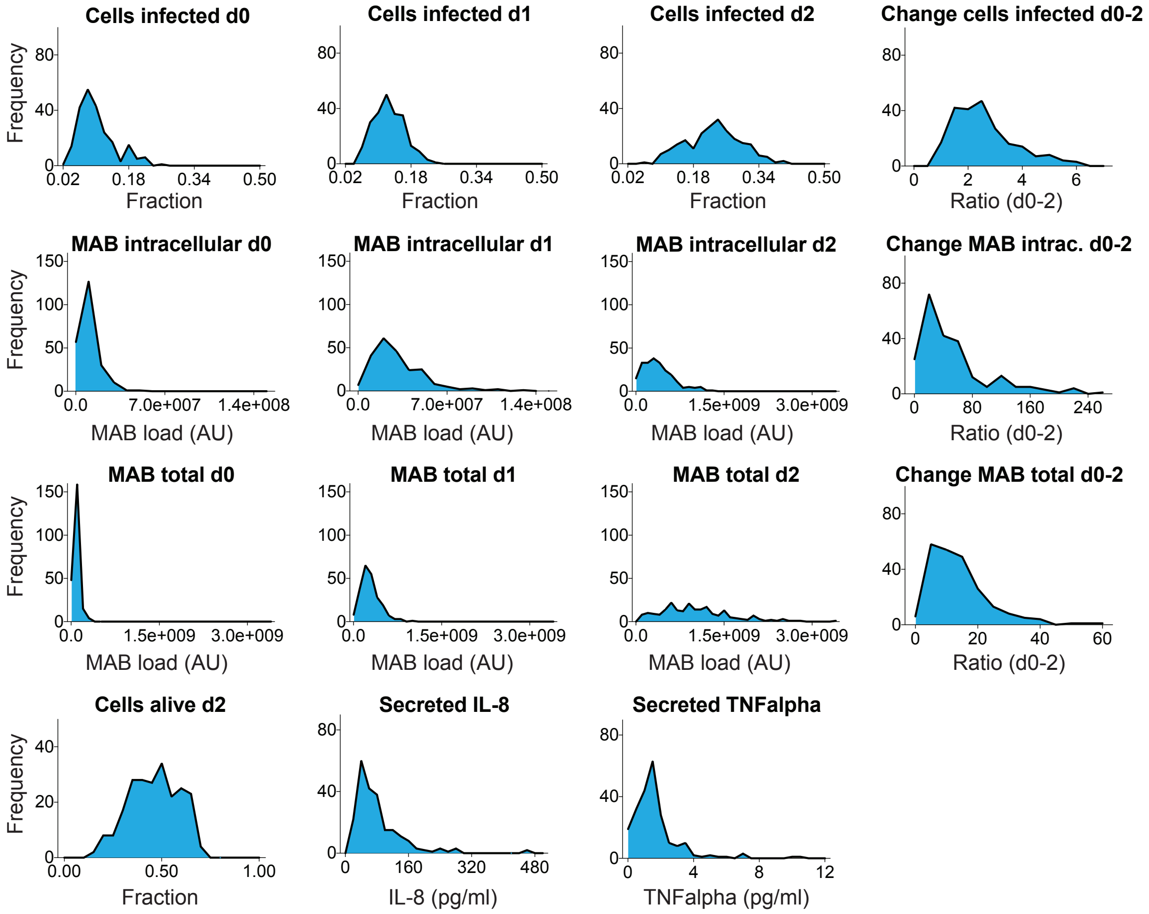

b. *In vivo* infection

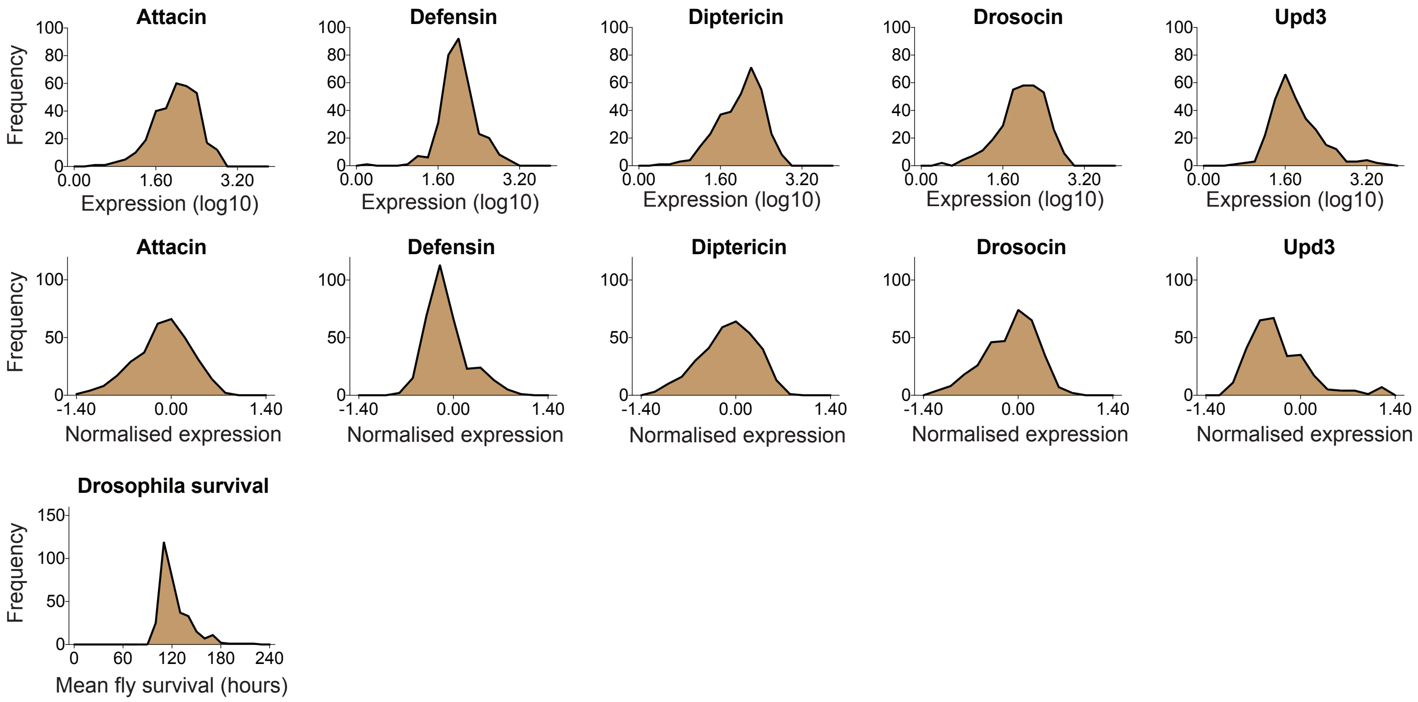

Supplementary Figure 3

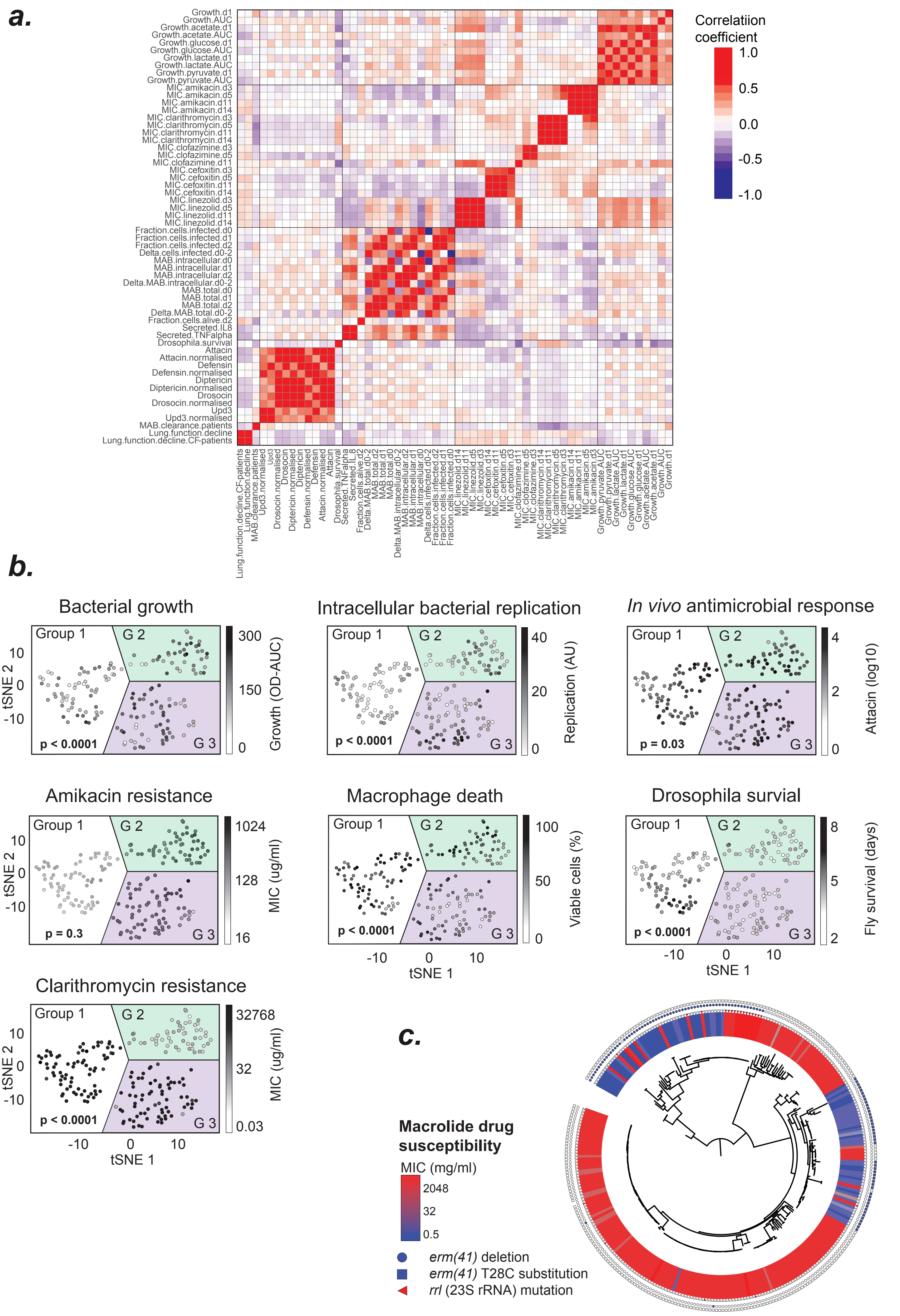

Supplementary Figure 4

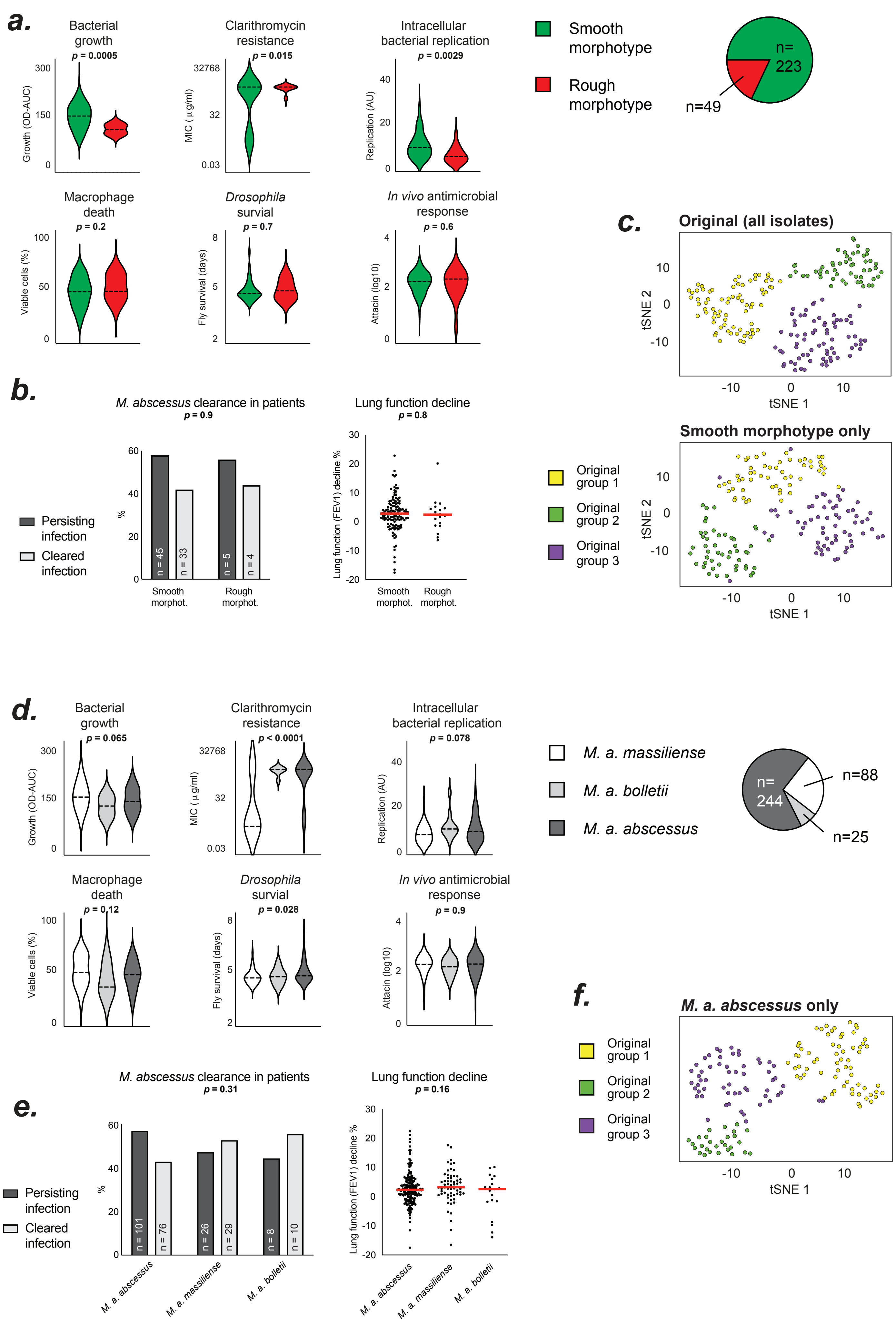

Supplementary Figure 5

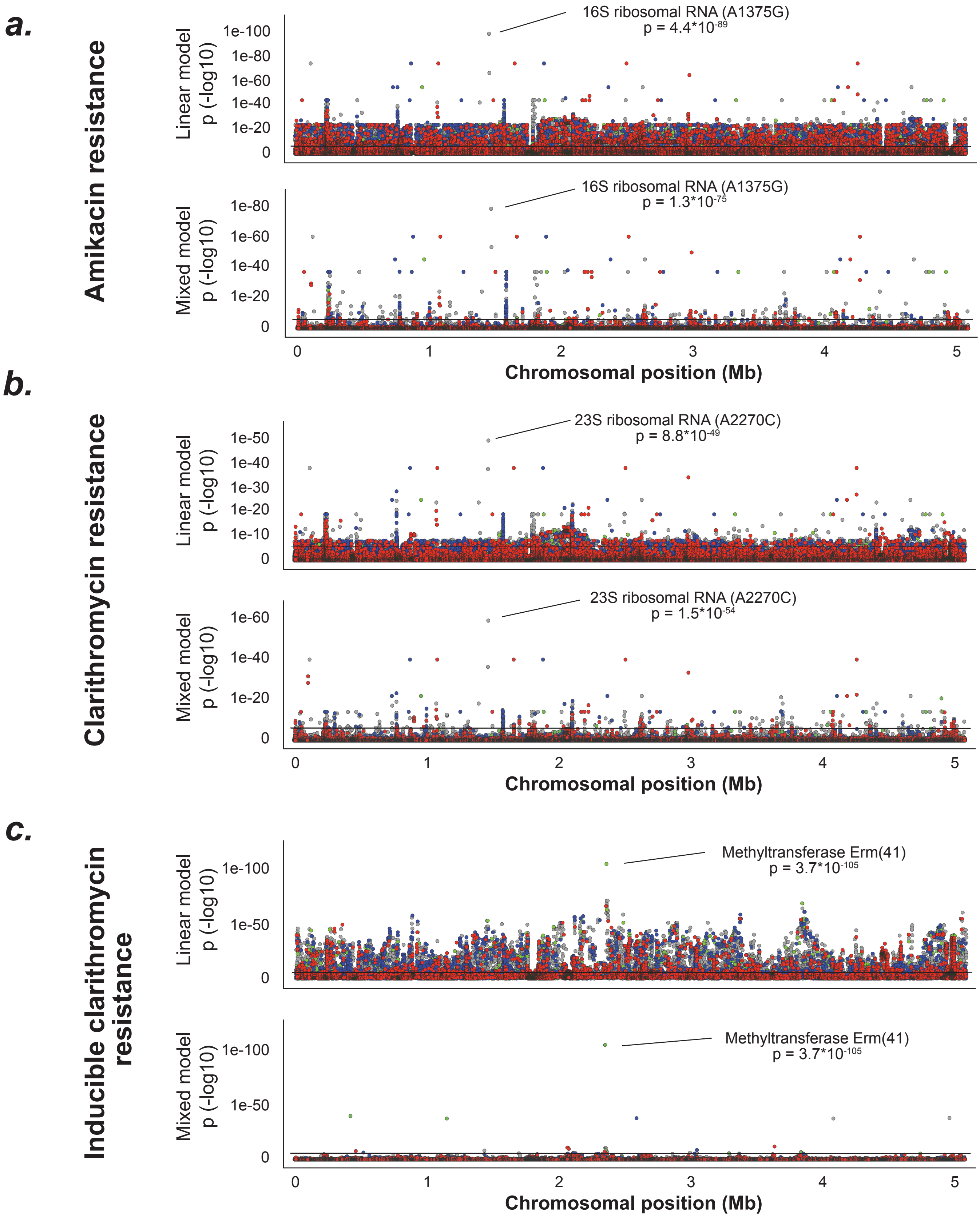

Supplementary Figure 6

a.

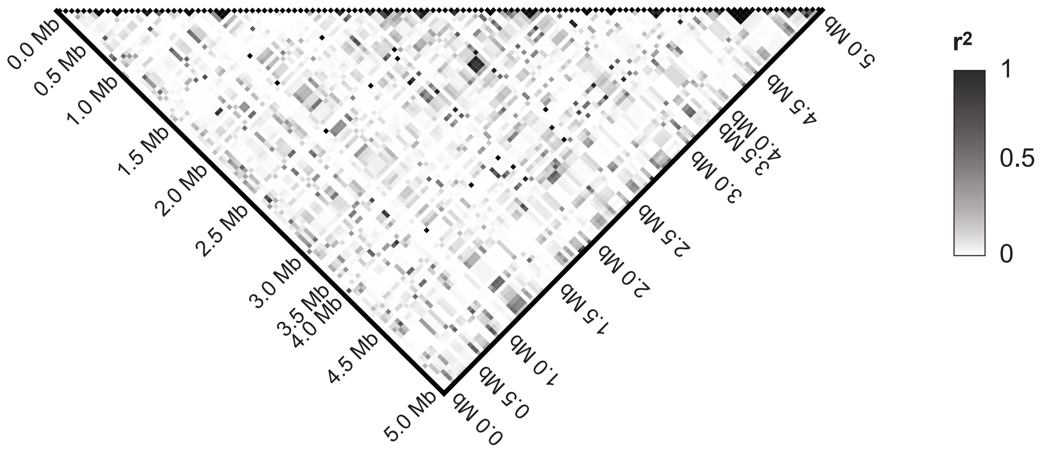

b.

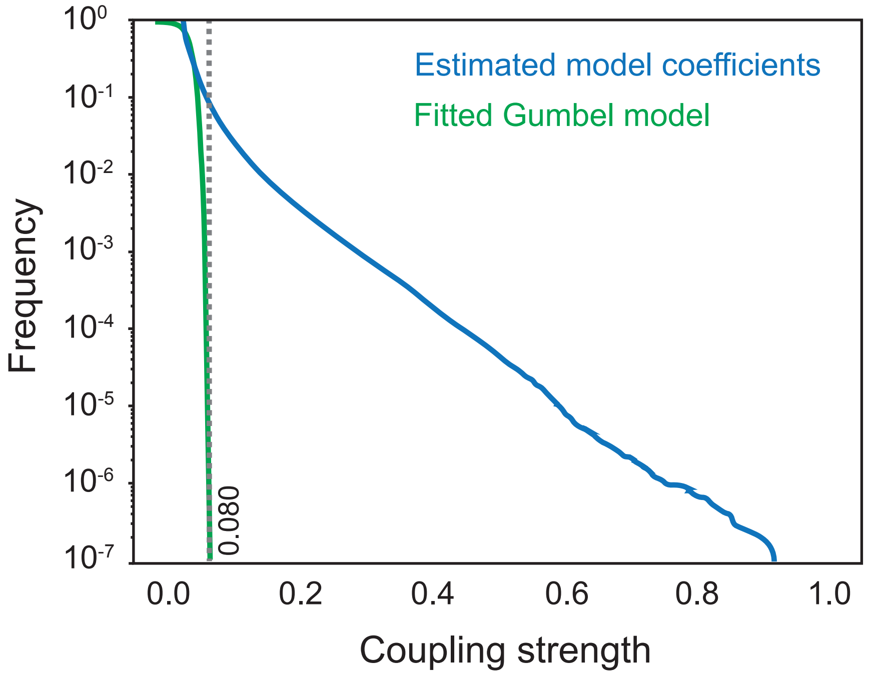

Supplementary Figure 7

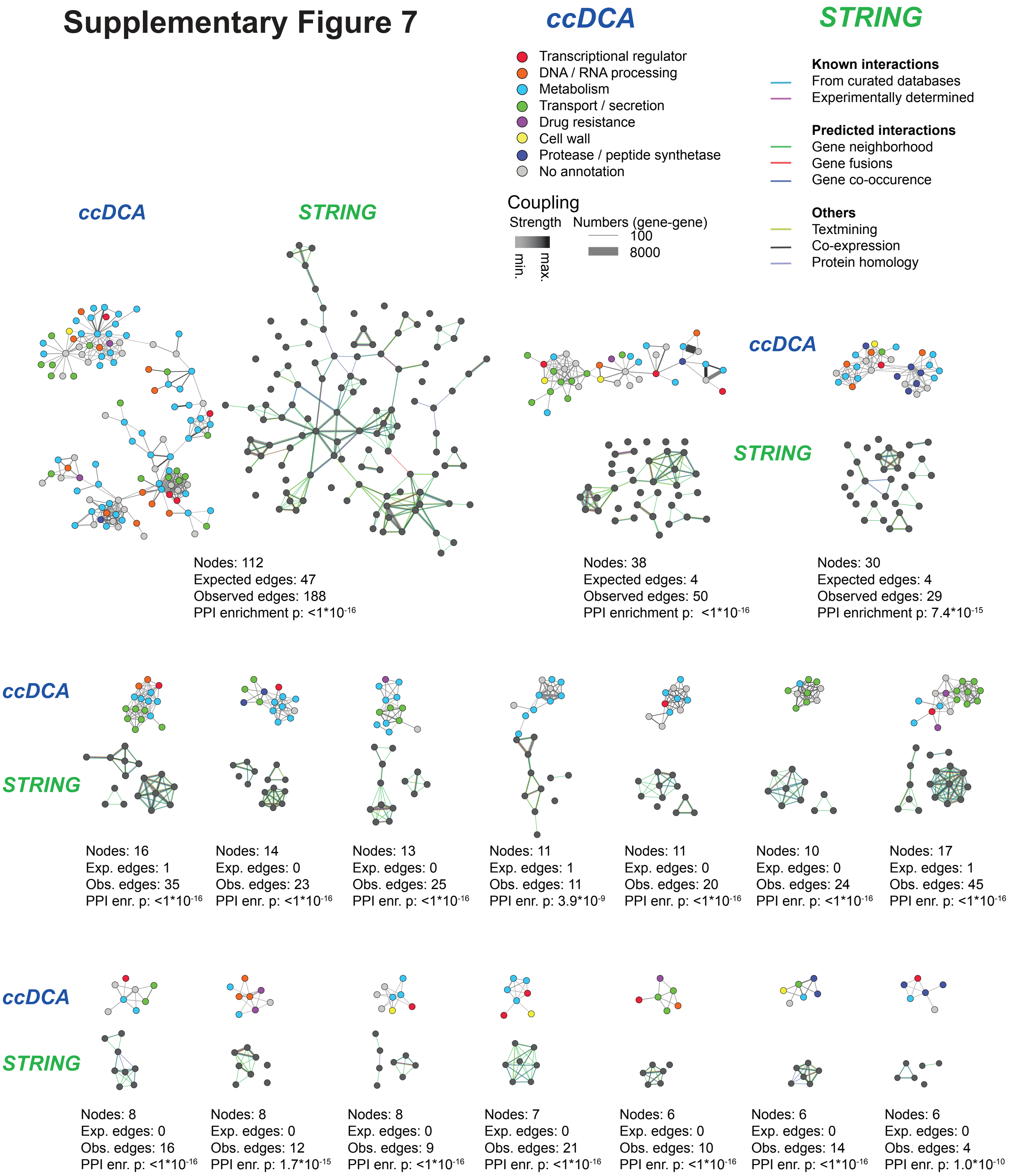

Supplementary Figure 8

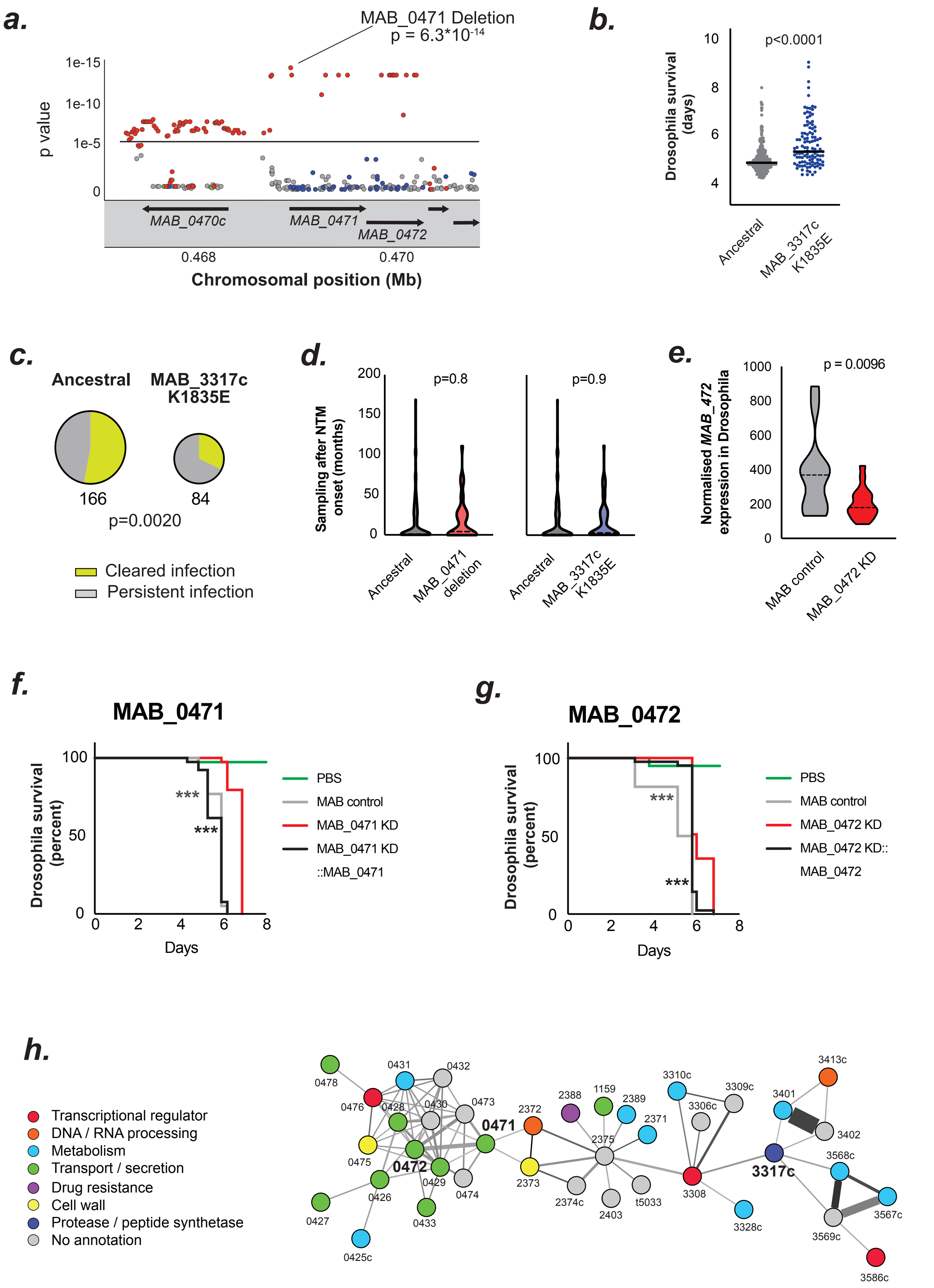

Supplementary Figure 9

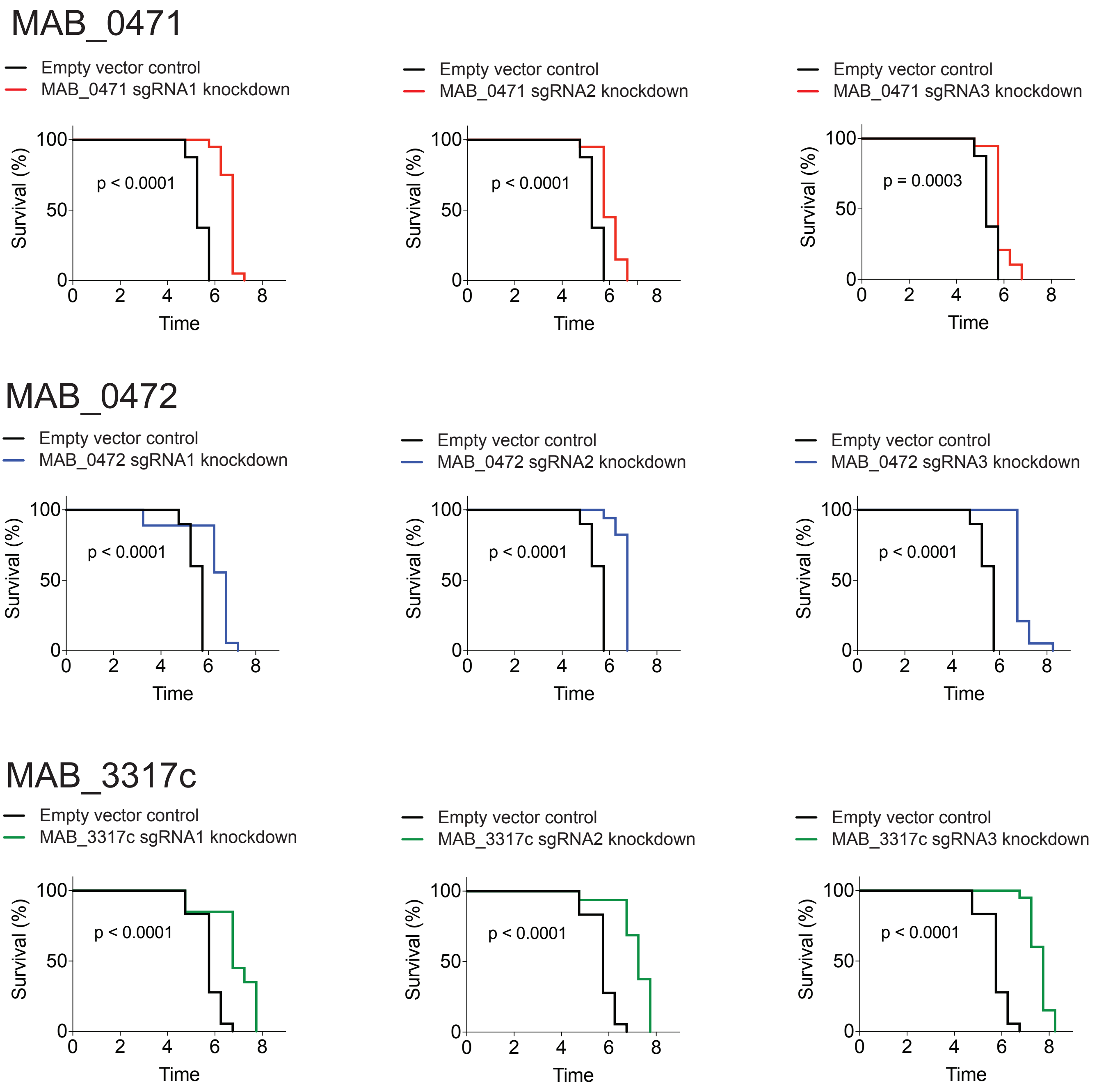

Supplementary Figure 10

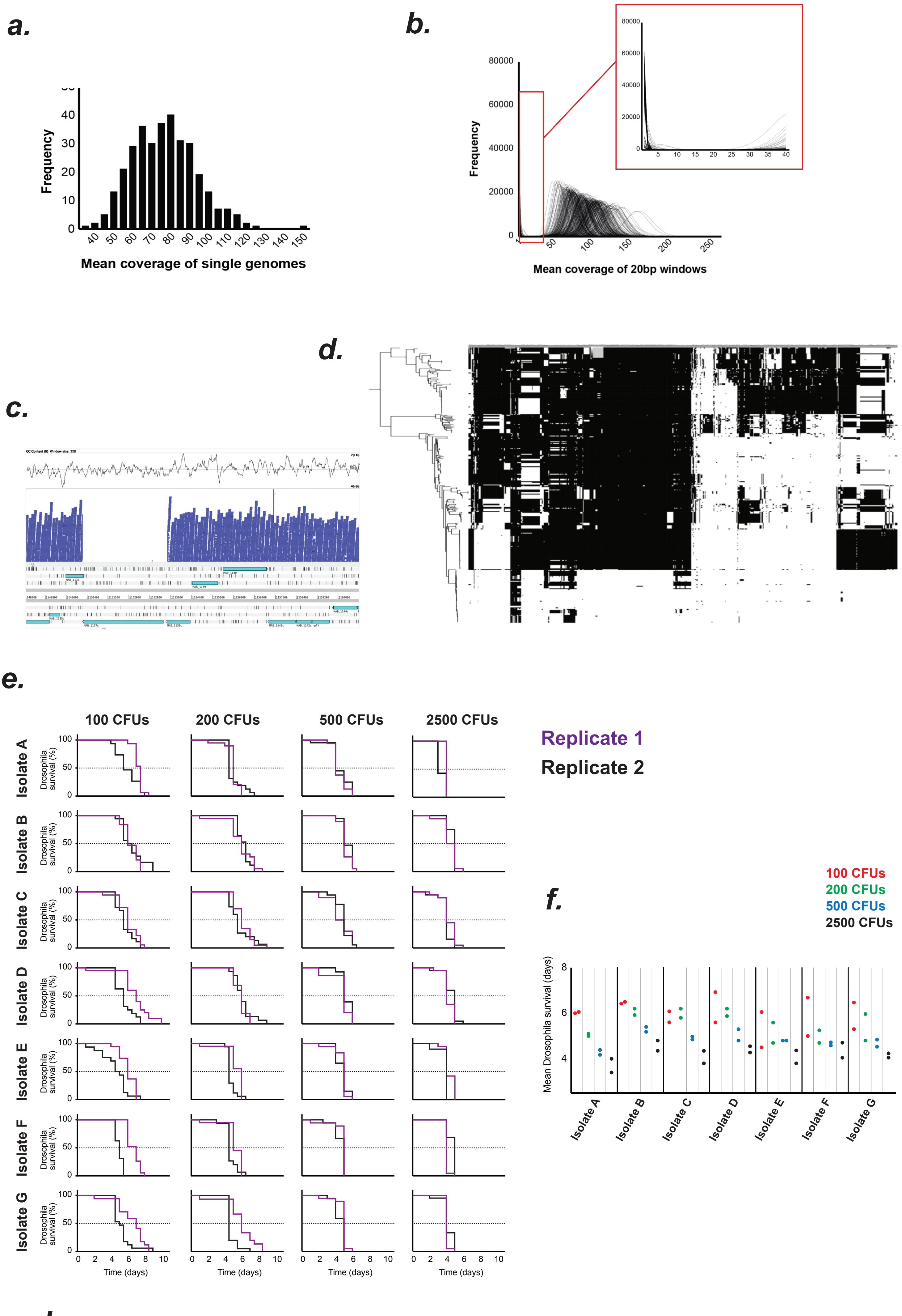

SUPPLEMENTARY TABLES

Supplementary Table 1: Patient baseline characteristics.

	n (with available data)	Median [interquartile range] or %
**Age (years)**	315	17 [13-23]
**Sex (female)**	319	42.0%
**Body mass index (kg/m2)**	291	21 [18-24]
**Cystic Fibrosis**	275	96.3%
**ATS defined NTM lung disease**	312	85.0%
**Sampling after NTM onset (months)**	158	1.2 [0.5-26.8]
**Clearance of NTM infection**	296	50.3%
**Lung function change within 1 year (% FEV1 decline)**	276	2.8 [0.1-5.6]

Supplementary Table 2: qPCR primer sequences.

Gene (*Drosophila*)	Primer 1	Primer 2
Attacin	CACAATGTGGTGGGTCAGG	GGCACCATGACCAGCATT
Defensin	TTCTCGTGGCTATCGCTTTT	GGAGAGTAGGTCGCATGTGG
Diptericin	ACCGCAGTACCCACTCAATC	CCCAAGTGCTGTCCATATCC
Drosocin	CCATCGAGGATCACCTGACT	CTTTAGGCGGGCAGAATG
Rpl1	TCCACCTTGAAGAAGGGCTA	TTGCGGATCTCCTCAGACTT
Upd3	ACTGGGAGAACACCTGCAAT	GCCCGTTTGGTTCTGTAGAT
**Gene (*M. abscessus*)**	**Primer 1**	**Primer 2**
MAB_0472	CCATCGGCTTCCTGCTTCTT	CCGAACATGTCGAGGCTGAA
	**Probe**	
	TCGAGCGGGACGACATCGAA	

Supplementary Table 3: Oligonucleotide sequences.

Gene	CRISPR guide Nr.	Forward	Reverse
YidC		CGCTGGGCCCCAAGAACTTCTCATG	AGAAGTTCTTGGGGCCCAG
MAB_471	1	CGGAGTGCGCTGGTGCTGACGGCATG	CCGTCAGCACCAGCGCACTC
MAB_471	2	CGGATCTCGCGTCGAATCGGAGCATG	CTCCGATTCGACGCGAGATC
MAB_471	3	CGAAGGTATCGGCGGCTGGACCCATG	GGTCCAGCCGCCGATACCTT
MAB_472	1	CGGGCTTCCTGCTTCTTTCGAGCATG	CTCGAAAGAAGCAGGAAGCC
MAB_472	2	CGTTCTTTCGAGCGGGACGACACATG	TGTCGTCCCGCTCGAAAGAA
MAB_472	3	CGACATGTTCGGTGTGTGTCTGCATG	CAGACACACACCGAACATGT
MAB_3317c	1	CGCCGGACATTGTCGTCGTTGGCATG	CCAACGACGACAATGTCCGG
MAB_3317c	2	CGACACGATGCCGTCGATTCCTCATG	AGGAATCGACGGCATCGTGT
MAB_3317c	3	CGCGGTGCTGAAAGAGTGCCCGCATG	CGGGCACTCTTTCAGCACCG

Gene	Mutant	Forward oligo	Reverse oligo
MbtD	mut256	CCGAGGACGGTGTGTTCGCG	TGAGGTGCTCGACGATTTGGTGAACC
MbtD	mut410	GCCGAGGCACCCGACCGGGTAT	GCGTAGGGCATCCCAGTTGTAGTCCAG

Supplementary Table 4: Reagents.

Acetate solution	Sigma-Aldrich	3863
Amikacin sulfate	Sigma-Aldrich	A1774
Anhydrotetracycline	Cayman	10009542
Cefoxitin	Sigma-Aldrich	C4786
Cellmask	Invitrogen	C10046
Clarithromycin	Sigma-Aldrich	C9742
Clofazimine	Sigma-Aldrich	C8895
DAPI	Thermo Fisher Scientific	62248
DMEM	Sigma-Aldrich	D6429
Fetal Bovine Serum	Thermo Fisher Scientific	10082147
Glucose	Sigma-Aldrich	G8270
Glycerol	Thermo Fisher Scientific	G/0600/08
Hygromycin B	Sigma-Aldrich	400051
Kanamycin Sulfate	Sigma-Aldrich	420411
Linezolid	Sigma-Aldrich	A10533
Middlebrook 7H11 Agar	Sigma-Aldrich	M0428
Middlebrook 7H9 Broth	Sigma-Aldrich	M0178
Middlebrook ADC	Sigma-Aldrich	M0553
Middlebroook OADC	Sigma-Aldrich	M0678
Mueller Hinton Broth	Thermo Fisher Scientific	YT3462
Penicillin Streptomycin	P4333	P4333
Phorbol-12-myristat-13-aceta	Sigma-Aldrich	P1585
qPCRBIO Probe Mix Lo-ROX	PCR Biosystems	PB20.11-05
RevertAid Reverse Transcriptase	Thermo Fisher Scientific	EP0441
RPMI 1640 with UltraGlutamine I	Lonza	BE12-702F
Sensimix™ SYBR no-ROX kit	Bioline	QT650-05-BL
Sodium L-lactate	Sigma-Aldrich	L7022
Sodium pyruvate	Sigma-Aldrich	12539059
TRIzol	Thermo Fisher Scientific	15596018
Tweeen 80	Sigma-Aldrich	P4780
Zeocin	AlfaAesar	J67140.8=
