# Supplementary material for: Mycobacterium abscessus pathogenesis identified by phenogenomic analyses
Source: Nat Microbiol. 2022 Aug 25;7(9):1431–41. doi: 10.1038/s41564-022-01204-x (PMC9418003; doi:10.1038/s41564-022-01204-x)
Supplement: Supplementary file 2 — Reporting Summary [file 41564_2022_1204_MOESM2_ESM.pdf]

nature portfolio

Corresponding author(s): R. Andres Floto

Last updated by author(s): 06/13/2022

Reporting Summary

Nature Portfolio wishes to improve the reproducibility of the work that we publish. This form provides structure for consistency and transparency in reporting. For further information on Nature Portfolio policies, see our Editorial Policies and the Editorial Policy Checklist.

Statistics

For all statistical analyses, confirm that the following items are present in the figure legend, table legend, main text, or Methods section.

n/a	Confirmed	
☐	☒	The exact sample size (*n*) for each experimental group/condition, given as a discrete number and unit of measurement
☐	☒	A statement on whether measurements were taken from distinct samples or whether the same sample was measured repeatedly
☐	☒	The statistical test(s) used AND whether they are one- or two-sided<br>*Only common tests should be described solely by name; describe more complex techniques in the Methods section.*
☒	☐	A description of all covariates tested
☐	☒	A description of any assumptions or corrections, such as tests of normality and adjustment for multiple comparisons
☐	☒	A full description of the statistical parameters including central tendency (e.g. means) or other basic estimates (e.g. regression coefficient) AND variation (e.g. standard deviation) or associated estimates of uncertainty (e.g. confidence intervals)
☐	☒	For null hypothesis testing, the test statistic (e.g. F, t, r) with confidence intervals, effect sizes, degrees of freedom and P value noted<br>*Give P values as exact values whenever suitable.*
☒	☐	For Bayesian analysis, information on the choice of priors and Markov chain Monte Carlo settings
☐	☒	For hierarchical and complex designs, identification of the appropriate level for tests and full reporting of outcomes
☐	☒	Estimates of effect sizes (e.g. Cohen's *d*, Pearson's *r*), indicating how they were calculated

Our web collection on statistics for biologists contains articles on many of the points above.

Software and code

Policy information about availability of computer code

Data collection	For data collection no software was used.
Data analysis	For data analysis the following software was used: BWA 0.7.13, Bcftools 1.7, SNPeff 4.3, sambamba 0.6.7, fastTree 2.1.11, itol v5, PLINK 1.7, Gemma 0.98, GEC 1.0, Columbus (2.9.0, Perkin Elmer), Rtsne 0.15, R Growthcurver 4.0, LocusZoom 1.4, Mabellini, HH-suite3, Hhsearch, MODELLER 9.12, mCSM, CC-DCA, Rosetta 3.11, Circos 0.69.8, Cytoscape 3.8.2, STRING v11.5

For manuscripts utilizing custom algorithms or software that are central to the research but not yet described in published literature, software must be made available to editors and reviewers. We strongly encourage code deposition in a community repository (e.g. GitHub). See the Nature Portfolio guidelines for submitting code & software for further information.

Data

Policy information about availability of data

All manuscripts must include a data availability statement. This statement should provide the following information, where applicable:

- Accession codes, unique identifiers, or web links for publicly available datasets
- A description of any restrictions on data availability
- For clinical datasets or third party data, please ensure that the statement adheres to our policy

All sequencing data of this study is deposited in the European Nucleotide Archive with the respective accession codes provided in Supplementary Table 6. Source data are provided with this paper.

Field-specific reporting

Please select the one below that is the best fit for your research. If you are not sure, read the appropriate sections before making your selection.

☒ Life sciences ☐ Behavioural & social sciences ☐ Ecological, evolutionary & environmental sciences

For a reference copy of the document with all sections, see nature.com/documents/nr-reporting-summary-flat.pdf

Life sciences study design

All studies must disclose on these points even when the disclosure is negative.

Sample size	The required GWAS sample sizes were based on assumed effect sizes of antimicrobial resistance and the number of available samples. With this sample sizes we could identify several unknown mechanisms; however it is likely that a much larger data set (n>1000) would have revealed even more information.
Data exclusions	M. abscessus isolates were phenotyped in replicates. If replicate variation was too large (as outlined in the online supplement), the phenotypic information was removed from final analysis.
Replication	Mycobacterial phenotyping was done in replicates and all replicates were analysed, except those not meeting quality criteria (as outlined in the online Supplement).
Randomization	Not applicable. Samples were not allocated to experimental groups.
Blinding	Not applicable. Samples were not allocated to experimental groups.

Reporting for specific materials, systems and methods

We require information from authors about some types of materials, experimental systems and methods used in many studies. Here, indicate whether each material, system or method listed is relevant to your study. If you are not sure if a list item applies to your research, read the appropriate section before selecting a response.

Materials & experimental systems

n/a	Involved in the study	
☒	☐	Antibodies
☐	☒	Eukaryotic cell lines
☒	☐	Palaeontology and archaeology
☐	☒	Animals and other organisms
☒	☐	Human research participants
☐	☒	Clinical data
☒	☐	Dual use research of concern

Methods

n/a	Involved in the study	
☒	☐	ChIP-seq
☒	☐	Flow cytometry
☒	☐	MRI-based neuroimaging

Eukaryotic cell lines

Policy information about cell lines

Cell line source(s)	ATCC TIB-202 (THP-1) was purchased direct from ATCC by us
Authentication	The cell line was not authenticated by us.
Mycoplasma contamination	Mycoplasma contamination was ruled out on a monthly base.
Commonly misidentified lines (See ICLAC register)	No misidentified cell lines were used in the study.

Animals and other organisms

Policy information about studies involving animals; ARRIVE guidelines recommended for reporting animal research

Laboratory animals	Drosophila melanogaster (w1118), male 6-8 day old
Wild animals	No wild animals were used in the study.
Field-collected samples	No field-collected samples were used in the study.

Ethics oversight	No ethical approval was required for the Drosophila work. All SOPs approved by Imperial College.

Note that full information on the approval of the study protocol must also be provided in the manuscript.

Clinical data

Policy information about clinical studies

All manuscripts should comply with the ICMJE guidelines for publication of clinical research and a completed CONSORT checklist must be included with all submissions.

Clinical trial registration	N/A
Study protocol	N.A
Data collection	Stored patient samples (bacterial isolates) and clinical metadata were retrospectively collected. 300 patients with chronic respiratory conditions (cystic fibrosis) and pulmonary Mycobacterium abscessus infection. Baseline characteristics are outlined in Supplementary Table 5. Retrospective clinical metatdata of patients assessed during routine clinical assessments was used. No patient was recruited for this study. Ethical approval to use clinical metadata was obtained from the National Research Ethics Service (NRES; REC reference: 12/EE/0158) and the National Information Governance Board (NIGB; ECC 3-03 (f)/2012) for centres in England and Wales; from NHS Scotland Multiple Board Caldicott Guardian Approval (NHS Tayside AR/SW) for Scottish centres; and respective review boards from Queensland (Australia) and the University of North Carolina (USA).
Outcomes	Patients were classified as having cleared M. abscessus infection (defined as documented culture conversion or a sustained clinical improvement where further cultures were unavailable) or as having persistent infection (if cultures remained positive or the clinical state worsened where no cultures were available).<br>Lung function decline was estimated as the percentage change in the forced expiratory volume (FEV1) from the available lung function assessment over a period of 12 months from baseline (before infection).
